# Distance-dependent spatial analysis of micropattern-generated shockwave for cell-type specific intracellular delivery

**DOI:** 10.1007/s10544-025-00758-x

**Published:** 2025-06-23

**Authors:** Aniket Mishra, Shunya Okamoto, Takayuki Shibata, Tuhin Subhra Santra, Sangjin Ryu, Moeto Nagai

**Affiliations:** 1 Department of Mechanical Engineering, Toyohashi University of Technology, Toyohashi, Japan; 2 Department of Engineering Design, Indian Institute of Technology, Madras, India; 3 Department of Mechanical and Materials Engineering, University of Nebraska-Lincoln, Lincoln, NE USA; 4 Nebraska Center for Materials and Nanoscience, University of Nebraska-Lincoln, Lincoln, NE USA; 5 Institute for Research on Next-generation Semiconductor and Sensing Science (IRES²), Toyohashi University of Technology, Toyohashi, Japan

**Keywords:** Intracellular delivery, Micropattern optoporation, Shockwave, Bioeffects, Cell poration

## Abstract

Intracellular delivery of therapeutic materials remains challenging, with conventional micropattern-assisted optoporation methods making it difficult to analyze the spatial effects of individual laser pulses. Here, we show that pigmented SU-8 microdisks enable precise analysis of distance-dependent shockwave effects on cell membrane permeabilization, achieving delivery yields up to 60% in optimized conditions. Using 20 μm and 50 μm microdisks irradiated by nanosecond laser pulses, we discovered that larger patterns generate more extensive shockwaves leading to increased cell damage over broader ranges, while smaller patterns maintain high delivery efficiency with minimal cellular disruption. Furthermore, cellular adhesion strength critically influences treatment outcomes: strongly adherent SAOS-2 cells showed remarkable resilience while weakly adherent HEK-293 cells experienced extensive damage at greater distances. Our results demonstrate how micropattern size and cell-specific properties determine the spatial extent and efficiency of shockwave-mediated delivery, providing a framework for optimizing intracellular delivery strategies while preserving cell viability.

**Supplementary Information:**

The online version contains supplementary material available at 10.1007/s10544-025-00758-x.

## Introduction

Cellular function deteriorates with age, injury, and disease, necessitating the delivery of therapeutic agents like drugs and nucleic acids into cells for treatment. However, the cell membrane’s semi-permeable nature poses a major challenge to this delivery process. Two primary approaches have emerged to overcome this barrier: carrier-mediated delivery using biochemical compounds (Kesharwani et al. 2014; Naseri et al. 2015; Sharma et al. 2015; Yadav et al. 2017), and membrane-penetrating delivery employing physical methods (Yao et al. 2008, 2020; Zhang and Yu 2008; Liang et al. 2010; Soman et al. 2011; Santra et al. 2014, 2020; Wilson et al. 2018; Shinde et al. 2020). While carrier-mediated approaches utilize vehicles such as liposomes (Yadav et al. 2017), dendrimers (Kesharwani et al. 2014), microspheres (Sharma et al. 2015), and solid lipid nanoparticles(Naseri et al. 2015), these carriers can potentially induce cellular toxicity.

To reduce carrier-based toxicity, physical methods such as electroporation (Gehl 2003; Santra et al. 2014), microinjection (Zhang and Yu 2008), sonoporation (Liang et al. 2010), mechanoporation (Chakrabarty et al. 2022), hydroporation (Zhang et al. 2004; Kim et al. 2025) and optoporation (Yao et al. 2008, 2020; Soman et al. 2011; Xiong et al. 2016; Wilson et al. 2018; Shinde et al. 2020) have emerged as alternatives to carrier-based delivery systems while achieving membrane-penetrating intracellular delivery. Electroporation requires careful optimization of parameters including field duration, field strength, and pulse number to maintain cell viability. Excessive field strength or prolonged duration can prevent proper resealing of membrane pores, leading to cell death through electrolyte imbalance (Canatella et al. 2001; Weiss et al. 2021). During sonoporation, ultrasonic acoustic pressure waves generate cavitation bubbles, whose random nature and transient characteristics can lead to cell death (Liu et al. 2012). Additionally, microinjection techniques suffer from low throughput due to difficulties in parallelization (Zhang and Yu 2008). Mechanoporation utilizes viscoelastic shear flow to create non-contact pores on cells. Hydroporation, in the context of intracellular delivery, incorporates high-pressure hydrodynamic shear to achieve pores. Both techniques are dependent on flow within microfluidic devices. While effective for high-throughput applications, these flow-based techniques encounter difficulties in targeting specific sub-populations of cells. This inherently limits precise temporal and spatial control, making site-specific intracellular delivery problematic.

Laser-based optoporation enables high-throughput, non-invasive delivery of cargo materials into cells through light-induced transient pore formation in cell membranes (Xiong et al. 2016; Wang et al. 2021). This technique comprises two main approaches: direct laser optoporation and photosensitized optoporation. While direct laser optoporation can create membrane pores by controlling laser parameters such as wavelength, intensity, and irradiation duration (Du et al. 2018), its application is limited by low throughput and operational complexity due to the requirement for precise laser focusing on individual cell membranes.

Traditional photosensitized optoporation relies on photoabsorber-laser interactions to generate pores in cell membranes. When laser light irradiates the photoabsorber material, vapor nanobubbles form at the cell medium-photoabsorber interface (Ramon et al. 2021). The growth and collapse of these bubbles create transient membrane pores. Conventional approaches typically employ gold or carbon nanoparticles attached to cell membranes through electrostatic attraction or antibody conjugation (Lapotko et al. 2009; Heinemann et al. 2014; Schomaker et al. 2015; Begandt et al. 2015; Kalies et al. 2015; Becker et al. 2019). While this nanoparticle-based approach effectively generates vapor nanobubbles, it presents two major challenges: difficulty in targeting specific cell populations and potential toxicity from the nanoparticles themselves (Eversole et al. 2020). Although researchers have attempted to address these limitations using modified photothermal substrates with different TiO_2_ nanostructures, such as nanoflowers and nanotubes (Mohan et al. 2021a, b), these substrate-based methods still fail to provide adequate spatial access for site-specific intracellular delivery.

Photolithographically patterned photo-absorbing micropatterns generate vapor nanobubbles upon pulsed laser irradiation through localized heating. Compared to traditional gold nanoparticle-based and modified substrate-based optoporation methods, micropattern-assisted optoporation provides precise targeting of specific sites with improved spatial control (Shinde et al. 2020, 2023). The versatility of micropatterns allows customization to specific shapes, offering advantages for heterogeneous cell populations (Nakashima et al. 2016; Deglincerti et al. 2016). While micropatterned devices have demonstrated high throughput optoporation of adherent cell lines (Shinde et al. 2020, 2023), previous approaches using titanium micropatterns as photoabsorbers faced significant limitations (Wu et al. 2015). The titanium-based method required complex vacuum deposition and time-consuming fabrication processes. Moreover, these devices needed to be inverted over cell monolayers during optoporation and subsequently removed after the procedure. This process compromised spatial information and hindered the tracking of specific target cells. Although delivery efficiency could be measured through flow cytometry or averaged image intensities, these methods inadequately captured the spatial dynamics of laser-micropattern interactions, leaving the distance-dependent effects of laser-induced cell perforation poorly characterized.

**Fig. 1:**
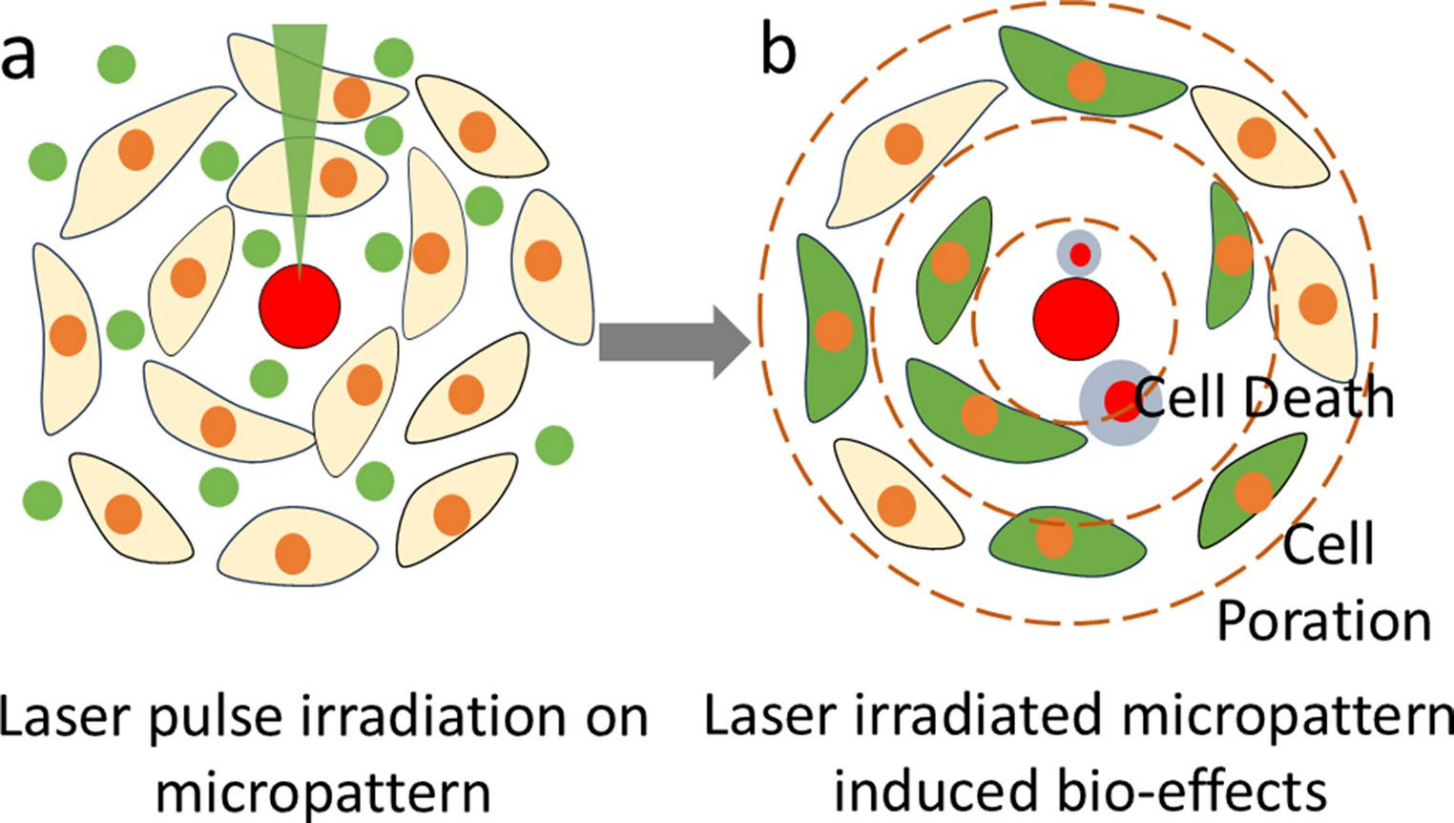
Representation of laser Irradiated micropattern induced bio effects

In this study, we developed an analytical method using pigmented SU-8 microdisks to investigate how laser-micropattern interactions affect cell membrane poration at varying distances (Fig. 1). This approach enables systematic characterization of spatial effects using different microdisk sizes and reveals cell type-specific responses to shockwave exposure. The simplified fabrication process combining spin-coating and photolithography allows direct cell culture adjacent to the patterns, facilitating precise spatial analysis of single-pulse shockwave effects on cell membranes.

## Experimental methods

### Fabrication of pigmented microdisks

0.45 g of red pigment, PR-254 (P1676, Tokyo Kasei, Japan) was mixed with 15 mL of cyclopentanone in a centrifuge tube. This mixture was then mixed with 15 mL of SU-8 3005 (KAYAKU Advanced Materials, USA) to obtain 1.5 w/v% of PR-254. The SU-8-PR-254 mixture was stirred for 20 min on a vortexer (TRIO HM-2 F, AZ-ONE, Japan) and then subjected to an ultrasonic cleaner (SonoCleaner 100D, KAIJO, Japan) for 5 min.

**Fig. 2:**
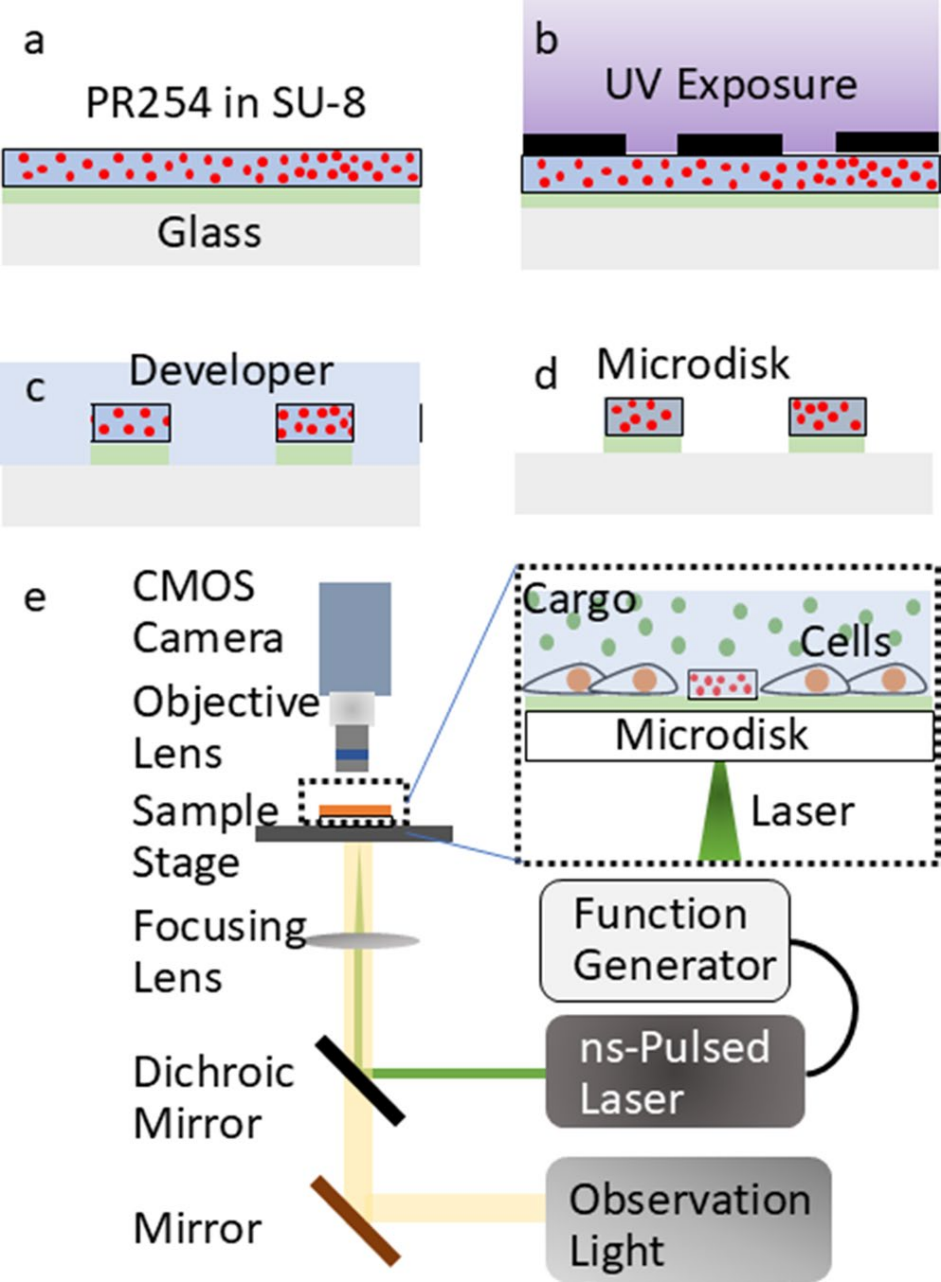
Schematic of microdisk fabrication and laser irradiation setup for the distance-dependent analysis method. (**a**)-(**d**) Steps in the fabrication of the pigmented SU-8 microdisk pattern. (**e**) Irradiation setup for optoporation, enabling precise control over shockwave magnitude and observation of cellular responses at different radial distances

The prepared mixture was spin-coated on a 4-inch glass wafer, and circular patterns with 20 and 50 μm diameters were formed by photolithography to obtain photo-absorbing disks (Fig. 2a-d). A 4-inch glass wafer was washed with acetone and rinsed with isopropanol (IPA), then dried with nitrogen gas. The substrate was plasma-treated using a plasma ashing system (JPA-300, J-Science Labs, Japan) at 150 W for 60 s to improve hydrophilicity. Hexamethyldisiloxane (HMDS) was spin-coated on the plasma-treated glass substrate with the following spin-coating steps: a gradual increase in speed for 5 s, 1000 rpm for 10 s, a gradual decrease in speed for 5 s, 5000 rpm for 30 s, a gradual decrease in speed for 10 s, and then baked. The suspension of SU-8-PR-254 was spin-coated on the HMDS layer at a slope for 5 s; 500 rpm for 20 s; slope for 5 s; 3000 rpm for 60 s; slope for 10 s. The wafer was baked again and exposed with a light integral of 600 mJ/cm^2^ using a mask aligner (PEM-800, Union Optical) with masks containing a 10 **×**10 array of 100 micropatterns of 50-µm diameter with 1-mm pitch and 12 array of 10 **×** 10 micropatterns of 20-µm diameter with 300-µm pitch. The wafer was baked again after exposure and developed in 2-methoxy-1-methylethyl acetate. The wafer was diced into small chips before cell culture, and a silicone wall structure was attached with bioadhesive glue to avoid slipping of cells from the device (Supplementary Fig. 1).

### Analysis of laser pulse and micropattern interaction

Micropattern chips with pattern diameters of 50 μm and 20 μm were filled with 200 µL of deionized (DI) water. To characterize bubble formation, a series of laser fluences (36, 43, 49, 51, 58, 126, 164, 200, and 514 mJ/cm²) were tested on 50 μm diameter microdisks. For each fluence level, 20 different microdisks were irradiated and bubble formation was recorded as a binary outcome (formed/not formed) using a CMOS camera (ZWO, ASL1600MC-Cool, Suzhou, China) with multi-dimensional acquisition in Micro-Manager 1.3 software. The probability of bubble formation was calculated as the percentage of successful formations out of 20 attempts.

For fluences that demonstrated 100% bubble formation probability (126–514 mJ/cm²), bubble size measurements were conducted. Images were acquired before and immediately after laser irradiation, and five independent measurements (*n* = 5) were performed for each fluence level on both 20 μm and 50 μm microdisk patterns. The acquired images were analyzed using ImageJ software to calculate the diameters of the resulting bubbles, and the mean diameter and standard deviation were determined from the five measurements.

### Cell culture on microdisks

A microdisk chip was sterilized with ethanol, acetone, and deionized (DI) water before cell culture. HeLa cells were cultured in minimum essential medium (MEM, Gibco, 11095072, Thermo Fisher Scientific, Waltham, MA, USA) supplemented with 10% fetal bovine serum (FBS, CCP-FBS-BR-500, Cosmo Bio, Tokyo, Japan) and 1% penicillin-streptomycin (streptomycin 10,000 U/mL, Thermo Fisher Scientific, Waltham, MA, USA). HEK293 and SAOS-2 cells were cultured in DMEM (FUJIFILM Wako Pure Chemical Co., Ltd., Tokyo, Japan) with 10% FBS and 1% penicillin–streptomycin (PS). The cells were grown in a polystyrene petri dish at 37 °C in a 5% CO_2_ humidified atmosphere. The adherent cultured cells were detached from the petri dish using trypsin EDTA. After replacement with fresh cell medium, the cells were seeded on the microchip at 37 °C with 5% CO_2_ in the air. Cells were then cultured to 80–90% confluency on the disk substrate and washed with PBS before laser irradiation.

### Optoporation and observation setup

We built a custom-made setup for laser-based optoporation (Fig. 2e). A nanosecond pulse laser (532 nm, Cobolt, TorTM XS, Hubner Photonics, Solna, Sweden) was connected to a function generator. A dichroic mirror (Thorlabs, DMLP550R, Newton, NJ, USA) was placed to reflect a 532-nm light beam. A convex lens with a focal length of 50 mm focused the laser beam on the sample with a spot diameter of around 140 μm. The sample was observed using an objective lens and CMOS camera (ZWO, ASL1600MC-Cool, Suzhou, China).

### Optoporation experiment, image acquisition, and analysis

A pigmented SU-8 chip was placed on the sample stage, and cell-impermeable FITC dextran (1 mM, Mw 4000 Da, Sigma Aldrich, USA) was added over the chip before laser irradiation. A single pulse with a duration of 5 ns was utilized for irradiating the microdisks. After laser irradiation, the cells were immediately washed three times with PBS and incubated in 4 µM PI for the viability assay.

Then micropattern substrate was placed on the stage of an inverted microscope (ECLIPSE Ti-2E, Nikon, Japan). The microscope was equipped with a camera (DS-Ri2, Nikon, Japan) and fluorescence cubes (B2-A and G-2 A Nikon, Japan). Exposure time was optimized for each sample while maintaining consistent exposure conditions within experimental group. Three images were obtained from each experiment and 5 experiments were performed. All reported delivery efficiency and cell viability data were obtained from the 15 images obtained from experiments. Area divisions of three concentric circles with radii of 40 μm, 80 μm, and 120 μm were drawn using python script. Then obtained images were processed with background correction with a rolling ball radius of 2 in ImageJ and the cells were manually counted using the multipoint tool of ImageJ software in all three regions (25–40 μm, 40–80 μm, and 80–120 μm) for 50 μm microdisk and (10–40 μm, 40–80 μm, and 80–120 μm) for 20 μm were counted to evaluate delivery efficiency, cell viability and delivery yield after treatment with the laser irradiation on micropattern.

The delivery efficiency *η*_*D*_, cell viability *η*_*V*_ and delivery yield *η*_*Y*_, were calculated from the total number of cells from brightfield images *N*_*T*_, the number of dye-positive cells *N*_*P*_, and the number of dead cells, *N*_*D*_. They were determined by the following Eqs. 1, 2 and 3:

1 $$\:{\eta\:}_{D}=\frac{{N}_{P}}{{N}_{T}}\:$$

2 $$\:{\eta\:}_{V}=\frac{\left({N}_{T}-{N}_{D}\right)}{{N}_{T}}\:$$

3 $$\:{\eta\:}_{Y}={\eta\:}_{P}{\eta\:}_{V}$$

Fluorescence appeared in the micropattern structure upon laser irradiation (Supplementary Figure S2). It was observed with/without cells. This fluorescence was excluded from cell counting and not further investigated.

### Statistical analysis

The delivery efficiency (*η*_D_), cell viability (*η*_V_), and delivery yield (*η*_Y_) were analyzed using JASP 0.19.0.0 software. First, the Shapiro-Wilk test was performed to assess data normality. Since some variables showed non-normal distributions, non-parametric tests were employed for all analyses. The Kruskal-Wallis test was conducted to evaluate differences between groups. When the Kruskal-Wallis test indicated significant differences (*p* < 0.05), Dunn’s post-hoc test with Bonferroni correction for multiple comparisons was performed. Statistical significance was set at *p*_*bonf*_ < 0.05.

## Experimental results

### Fabrication of optical absorber disks

**Fig. 3:**
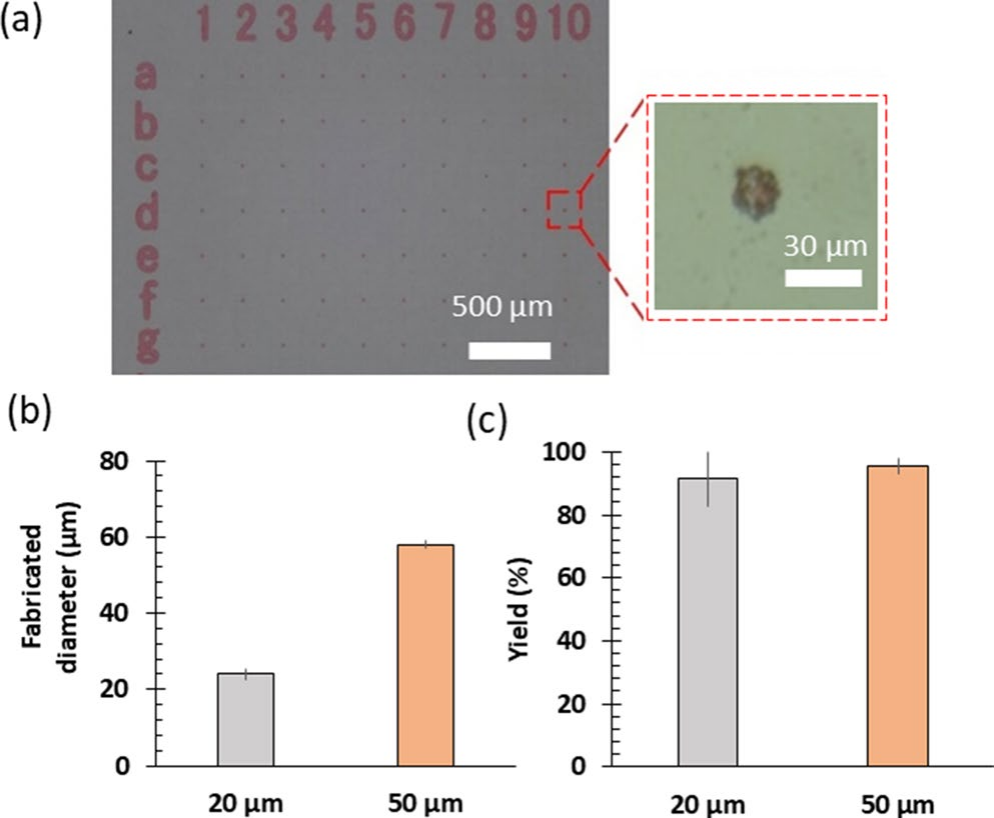
Fabricated pigmented microdisks. (**a**) Microscope image showing microdisks with 20 μm design diameters. (**b**) Fabricated diameters and (**c**) fabrication yields for 20 μm and 50 μm microdisk patterns

SU-8 microdisks containing PR-254 were formed on a glass wafer by photolithography. Micro-optical energy absorbers were patterned, and chips were obtained (Fig. 3a). For the 20 μm design diameter (D.D.), and the approximate fabricated diameter (F.D.) of the disks was 24 ± 1.5 μm (*n* = 25) (Fig. 3b) and 12 chips were obtained from one wafer with a pattern yield of 91.4 ± 8.60% (mean ± standard deviation, number of measured wafers: *n* = 6) (Fig. 3c). The microdisks with D.D. of 50 μm had an average F.D. of 57.5 ± 1.0 μm (*n* = 25) (Fig. 3b) and were obtained with an average yield of 95.5 ± 2.5% (number of measured wafers: *n* = 6) (Fig. 3c). Fabricated microdisks were found to be larger than the design value. With increasing D.D. size, the error increased. This difference in diameter was probably because the resolution of the film mask used in this experiment was limited, and the corners of the pattern were not fully transferred.

### Characterization of laser-induced bubble formation

**Fig. 4:**
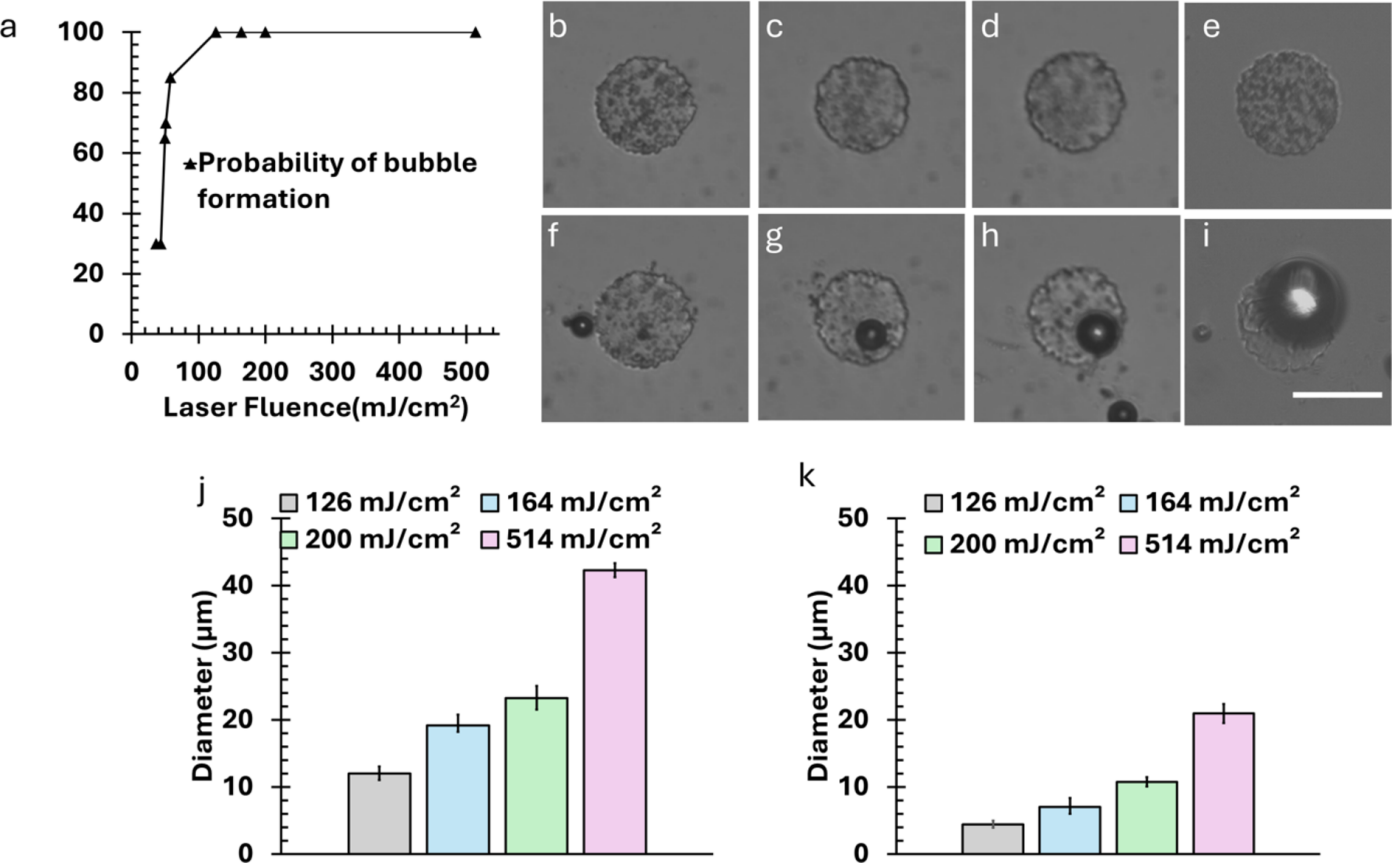
Bubble formation characteristics of microdisks. (**a**) Probability of bubble generation at various fluences for 50 μm microdisk. Images of 50 μm microdisk (**b**)–(**e**) before (**f**)–(**i**) after laser pulse irradiation 126 mJ/cm^2^, 164 mJ/cm^2^, 200 mJ/cm^2^, 514 mJ/cm^2^. Scale bar is 50 μm. (**j**) Bubble diameter measurement with 50-µm microdisk. (k) Bubble diameter measurement with 20 μm microdisk

We evaluated the probability of bubble formation at laser fluences of 36 mJ/cm^2^, 43 mJ/cm^2^, 49 mJ/cm^2^, 51 mJ/cm^2^, 58 mJ/cm^2^, 126 mJ/cm^2^, 164 mJ/cm^2^, 200 mJ/cm^2^, and 514 mJ/cm^2^ on the micropattern disks of 50 μm diameter. We measured the probability of bubble generation, which is indicated in Fig. 4(a) (*n* = 20 microdisks). Only laser fluences with 100% bubble formation probability were considered for further experiments measuring bubble size.

Figure 4(b)–(i) show the generation of larger bubble sizes with diameters of 50 μm and increasing laser fluences from 126 mJ/cm², 164 mJ/cm², 200 mJ/cm², to 514 mJ/cm². The 50-µm micropattern exhibited larger bubble sizes under the same energy conditions, with measurements of approximately 12.0 ± 1.0 μm, 19.2 ± 1.5 μm, 23.2 ± 1.7 μm, and 42.2 ± 1.0 μm (*n* = 5) (Fig. 4j). In contrast, for the 20 μm micropattern, the bubble sizes recorded were approximately 4.4 ± 0.4 μm, 7.0 ± 1.3 μm, 10.7 ± 0.6 μm, and 20.9 ± 1.4 μm (*n* = 5) as the energy increased from 126 mJ/cm² to 514 mJ/cm^2^ (Fig. 4k). Larger energy was absorbed in the 50-µm pattern, and allowed for greater accumulation of gas and subsequent bubble growth. The increase in energy input results in more vigorous gas generation and larger bubbles for both patterns, but the effect is more pronounced in the larger micropattern due to its enhanced capacity to sustain larger bubbles. Notably, at 514 mJ/cm^2^ laser fluence, we observed complete removal of the micropattern disc during some irradiation events. However, this removal was not consistent across all microdisks. The observed phenomenon is likely due to localized overheating or rapid gas expansion within the pattern, which occasionally disrupts the physical structure (Supplementary Fig. S3). This suggests that micropattern size plays a critical role in determining bubble dynamics during laser-induced processes and eventually contributes to the generation of poration effects on cell membrane.

### Optoporation of HeLa cells with 50 μm disk

**Fig. 5:**
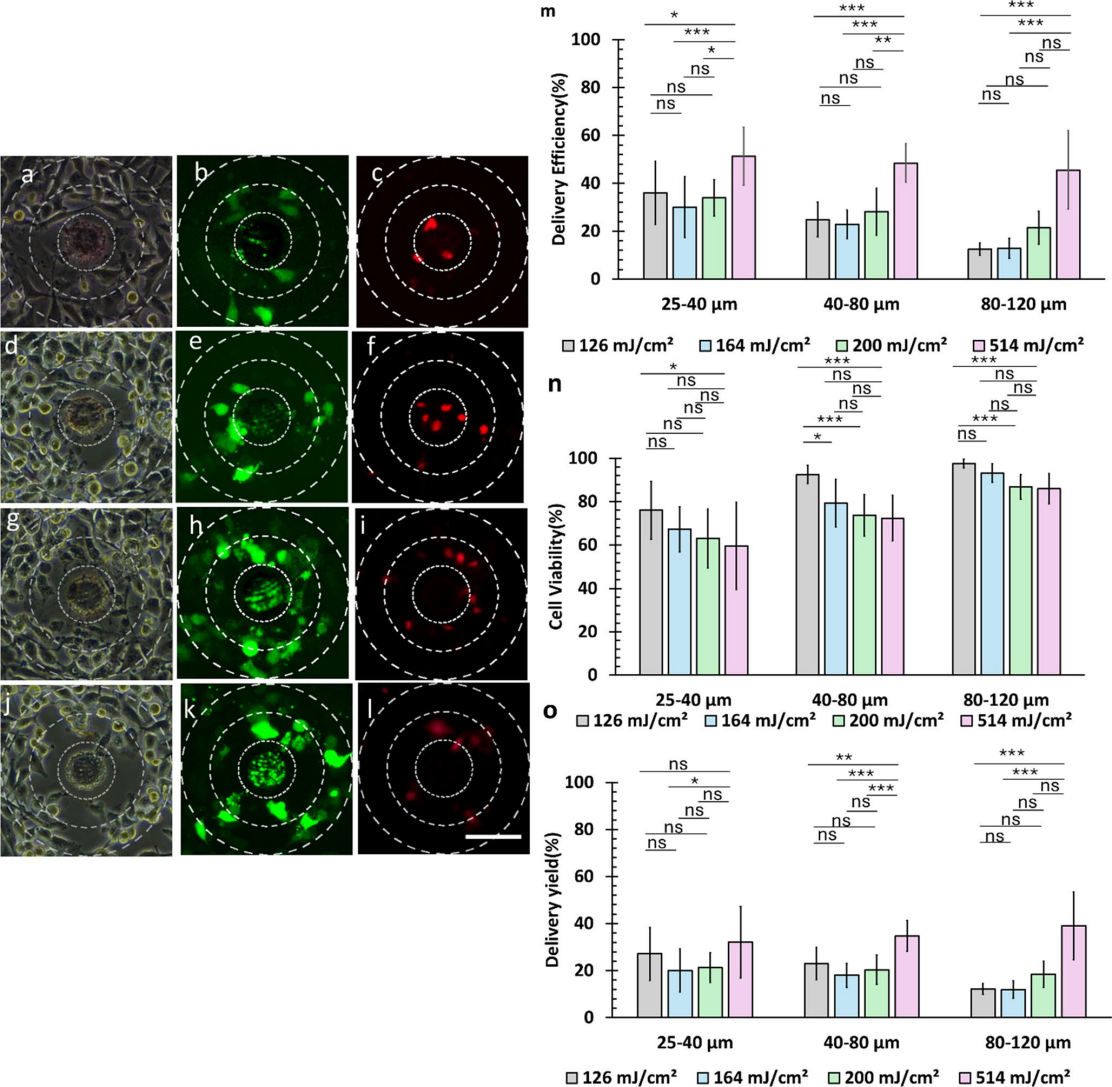
Laser fluence affects optoporation efficiency and cell viability in HeLa cells using a 50 μm diameter microdisk. (**a**–**l**) Representative images showing brightfield (left column), FITC-positive cells indicating successful delivery (green, middle column), and dead cells (red, right column) at different laser fluences. (**a**–**c**) 126 mJ/cm², (**d**–**f**) 164 mJ/cm², (g–i) 200 mJ/cm², and (**j**–**l**) 514 mJ/cm². Scale bar is 80 μm. (**m**) Delivery efficiency, (**n**) cell viability, and (**o**) delivery yield as a function of laser fluence and distance from the irradiation point

Figure 5 shows the effect of pattern size on micropattern-assisted optoporation with PR254 microdisks with diameters of 50 μm at laser fluences of 126 mJ/cm^2^, 164 mJ/cm^2^, 200 mJ/cm^2^, and 514 mJ/cm^2^. Figure 5(a)–(l) display fluorescence images of optoporated cells using 50 μm diameter disks. The laser irradiation of the 50 μm pattern resulted in intracellular delivery of FITC dextran 4 kDa into cells (Fig. 5b, e, h, k). Upon laser irradiation on the micropattern, cell viability was compromised in Fig. 5(c, f, i, l), indicating the influence of cell peeling or cell necrosis on the evaluated *η*_*D*_, *η*_*V*_, and *η*_*Y*_. No dead or optoporated cells were observed (Figure S4–S6) in the absence of laser irradiation of cells cultured on a micropatterned substrate, and in the absence of micropatterns with laser irradiation on a cell monolayer on a glass substrate.

Figures 5(m)–(o) show *η*_*D*_, *η*_*V*_, and *η*_*Y*_ with a 50-µm microdisk at various radial distances and laser fluences. Intuitively, *η*_*D*_ should be higher closer to the area of laser irradiation, but the calculation of delivery is affected by cells either peeling off or undergoing necrosis, affecting *η*_*Y*_ at a given fluence and radial distance. The cell response of HeLa cells to micropattern laser irradiation at 126 mJ/cm²(Supplementary video 1) and to 514 mJ/cm^2^ (Supplementary Video 2) visually demonstrate the difference in cell response at varying laser parameters.

The analysis of delivery yield (*η*_*Y*_) across different distances revealed complex relationships between delivery efficiency and laser fluence. In the 25–40 μm region, while the delivery efficiency at 514 mJ/cm² (51.3 ± 12.0%, average *N*_*T*_ =5 cells) was significantly higher than at 126 mJ/cm² (36.0 ± 13.2%, average *N*_*T*_ =7 cells, *p*_*bonf*_ =0.04), the final delivery yields remained statistically equivalent (32.0 ± 15.1% at 514 mJ/cm² versus 27.1 ± 11.3% at 126 mJ/cm², *p*_*bonf*_ =1). At greater distances with 514 mJ/cm², despite reduced cell viability, substantial delivery yields were maintained, reaching 34.7 ± 6.5% in the 40–80 μm region (delivery efficiency 48.4 ± 7.9%, average *N*_*T*_ =22 cells) and 39.07 ± 14.4% in the 80–120 μm region (delivery efficiency 45.5 ± 16.4%, average *N*_*T*_ =35 cells). These results demonstrate that while higher laser fluence initially produces greater delivery efficiency, the final yield remains consistent across distances due to the counterbalancing effects of cell viability.

Figure S7 shows an inverse relationship between distance from the laser irradiation point and cellular response: as distance increased, delivery efficiency (*η*_*D*_) decreased while cell viability (*η*_*V*_) increased. Using the 50 μm pattern at high laser fluence (514 mJ/cm²), uniform shockwave effects were observed throughout the 25–120 μm region, resulting in non-significant difference in yields from 25 to 40 μm to 40–80 μm(*p*_*bonf*_ =1), and from 40 to 80 μm to 80–120 μm (*p*_*bonf*_ =0.62).

At 200 mJ/cm², a clear trade-off emerged between delivery efficiency and cell viability in different regions. The 40–80 μm region exhibited higher delivery efficiency (28.1 ± 9.7%) despite lower cell viability (73.6 ± 9.5%), while the 80–120 μm region showed reduced delivery efficiency (21.4 ± 6.8%) but enhanced cell viability (86.7 ± 5.7%). These counterbalancing effects resulted in non-significant delivery yields (*p*_*bonf*_ =1) between regions. Lower laser fluences (164 mJ/cm² and 126 mJ/cm²) displayed similar patterns, with the 40–80 μm region achieving higher delivery efficiency but reduced viability compared to the 80–120 μm region.

Figure S7 reveals a key trade-off: as distance from the laser irradiation point increases, intracellular delivery efficiency decreases, while cell viability correspondingly increases. Consequently, regions closer to the irradiation site achieve higher delivery efficiency at the expense of lower cell viability, while regions farther away exhibit improved viability but lower efficiency. Despite these opposing trends, the final delivery yield remains comparable due to counterbalancing effects. From these observations, the 25–80 μm range emerges as the optimal zone, providing the most favorable balance between delivery and cell health. This finding highlights the critical need for spatial targeting to optimize optoporation performance.

### Optoporation of HeLa cells with 20 μm disk

**Fig. 6:**
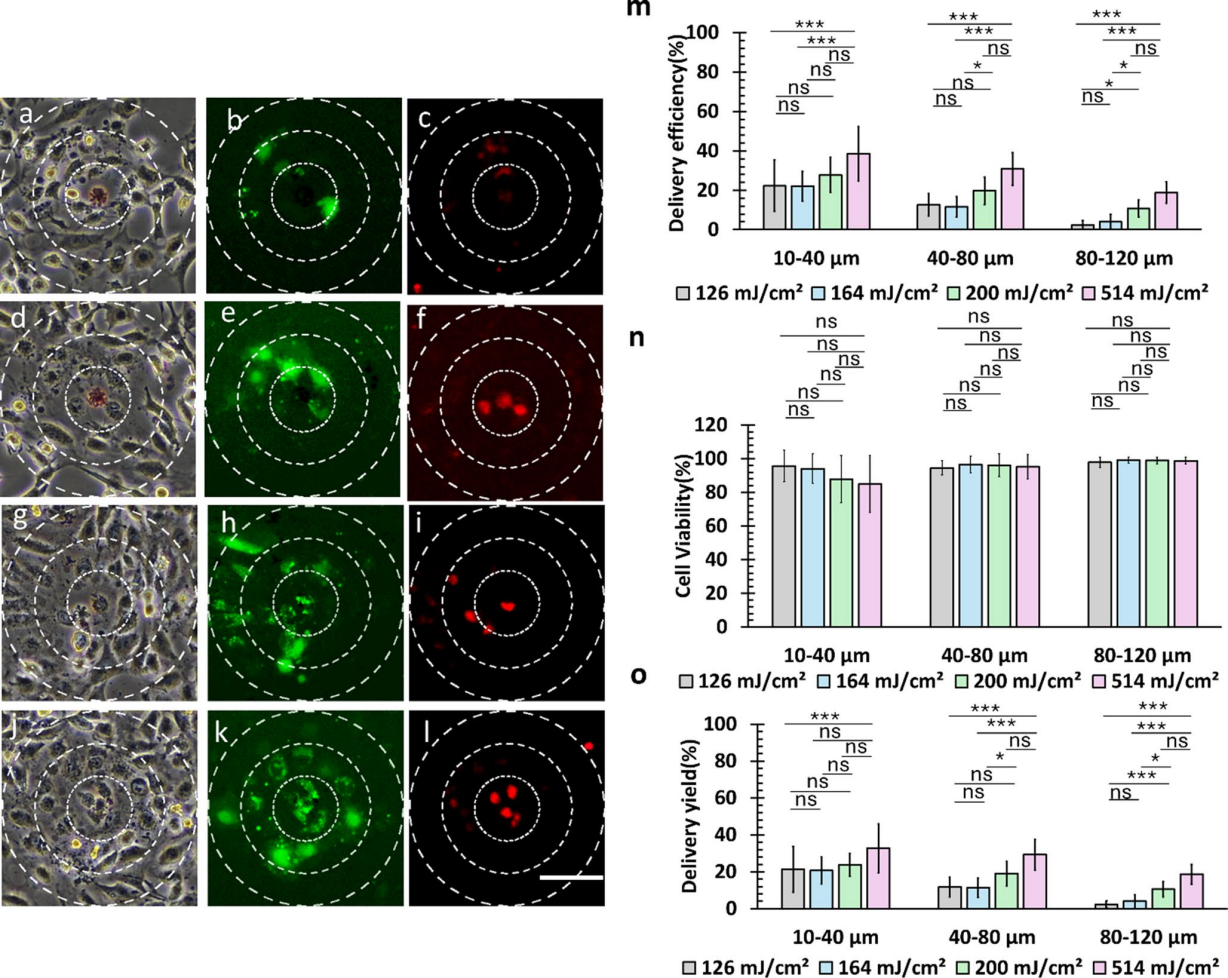
Optoporation of HeLa cells using a 20-µm-diameter microdisk at various laser fluences: (**a**-**c**) 126 mJ/cm², (**d**-**f**) 164 mJ/cm², (**g**-**i**) 200 mJ/cm², and (**j**-**l**) 514 mJ/cm². For each fluence, the left column shows brightfield images, the middle column shows FITC-positive cells (green, indicating successful delivery), and the right column shows dead cells (red). Scale bar: 80 μm. (**m**) Delivery efficiency, (**n**) cell viability, and (**o**) delivery yield measured as a function of laser fluence and distance from the irradiation point

The study revealed distinct differences in cellular responses between 20 μm and 50 μm microdisk patterns, with the smaller disk generating lower magnitude shockwaves across all measured distances (40 μm, 80 μm, and 120 μm). Fluorescence imaging demonstrated successful intracellular molecule delivery near the laser spot when using the 20 μm disk, achieving delivery patterns similar to those observed with the 50 μm pattern (Fig. 6b, e, h and k). Green fluorescence was observed near the laser spot from the disk.

Analysis of the 20-µm microdisk revealed distinct delivery characteristics compared to the 50-µm microdisk. Figures 6(m), 6(n), and 6(o) show *η*_*D*_, *η*_*V*_, and *η*_*Y*_, respectively, for the 20-µm microdisk pattern. Unlike the case with the 50-µm microdisk, *η*_*D*_ showed less impact from cell necrosis and peeling while maintaining higher cell viability. As suggested by the microbubble size patterns observed in the previous section, *η*_*D*_ increased proportionally with laser fluence.

In the range of 10–40 μm, the highest *η*_*Y*_ was obtained at a laser fluence of 514 mJ/cm^2^ (Fig. 6o), significantly exceeding the yield at 126 mJ/cm^2^ in the 10–40 μm region. This superior performance extended to the 40–80 μm region, where 514 mJ/cm^2^ demonstrated significantly higher yields compared to both 126 mJ/cm^2^ and 164 mJ/cm^2^, with similar results in the 80–120 μm range. A delivery yield of 32.6 ± 13.2% (average *N*_*T*_ = 6 cells) was obtained in the 10–40 μm range. The *η*_*Y*_ exhibited a distance-dependent decline, reaching 29.2 ± 8.3% at 40–80 μm (average *N*_*T*_ = 20 cells) and 18.5 ± 5.3% at 80–120 μm (average *N*_*T*_ = 34 cells) at 514 mJ/cm^2^.

Statistical analysis (Figure S8) revealed that at the same energy, delivery efficiency for laser fluences of 514 mJ/cm^2^ and 200 mJ/cm^2^ showed significant differences only between the 40–80 μm and 80–120 μm ranges (*p*_*bonf*_ = 0.008 for both fluences). At 164 mJ/cm^2^, significant differences were observed across all regions. At 126 mJ/cm^2^, only the regions between 40 and 80 μm and 80–120 μm showed highly significant differences (*p*_*bonf*_ = 0.002), likely due to minimal shockwave effects in the 80–120 μm regions.

Overall, the results from irradiation at the same energy indicate that the spatial extent and uniformity of laser-induced cellular effects are strongly influenced by laser fluence. At higher fluences, such as 514 and 200 mJ/cm², shockwave effects extend farther from the irradiation point, resulting in significant differences in delivery efficiency primarily between mid-range (40–80 μm) and far-range (80–120 μm) regions. In contrast, at lower fluences, these effects become more spatially restricted, with significant differences observed across all regions at 164 mJ/cm², and highly localized effects at 126 mJ/cm², where only the 40–80 μm and 80–120 μm regions differ significantly. These results indicate that higher laser fluence enhances the effective range of optoporation, enabling delivery to cells located farther from the micropattern, whereas lower fluence limits delivery to cells in closer proximity. Therefore, adjusting laser energy is crucial for controlling the spatial reach and uniformity of cellular response in optoporation applications.

A comparison between the 50-µm and 20-µm microdisk patterns revealed distinct impacts on cell response during optoporation, with each pattern showing unique distance-dependent characteristics. While the 50-µm microdisk pattern exhibited more aggressive effects through significant cell detachment and necrosis that impacted the overall delivery process, the 20-µm microdisk pattern demonstrated more controlled effects with proportionally increasing delivery efficiency at higher laser fluence while maintaining better cell viability. For cells within 40 μm of the irradiation site, the 20-µm microdisk achieved comparable delivery efficiencies with enhanced viability, whereas the 50-µm pattern proved more effective for targeting HeLa cells at distances beyond 40 μm, suggesting that pattern size selection should be optimized based on the specific application requirements, considering both target distance and the desired balance between delivery efficiency and cell viability.

### Optoporation of HEK-293 and SAOS-2 cells with 20 μm disk

**Fig. 7:**
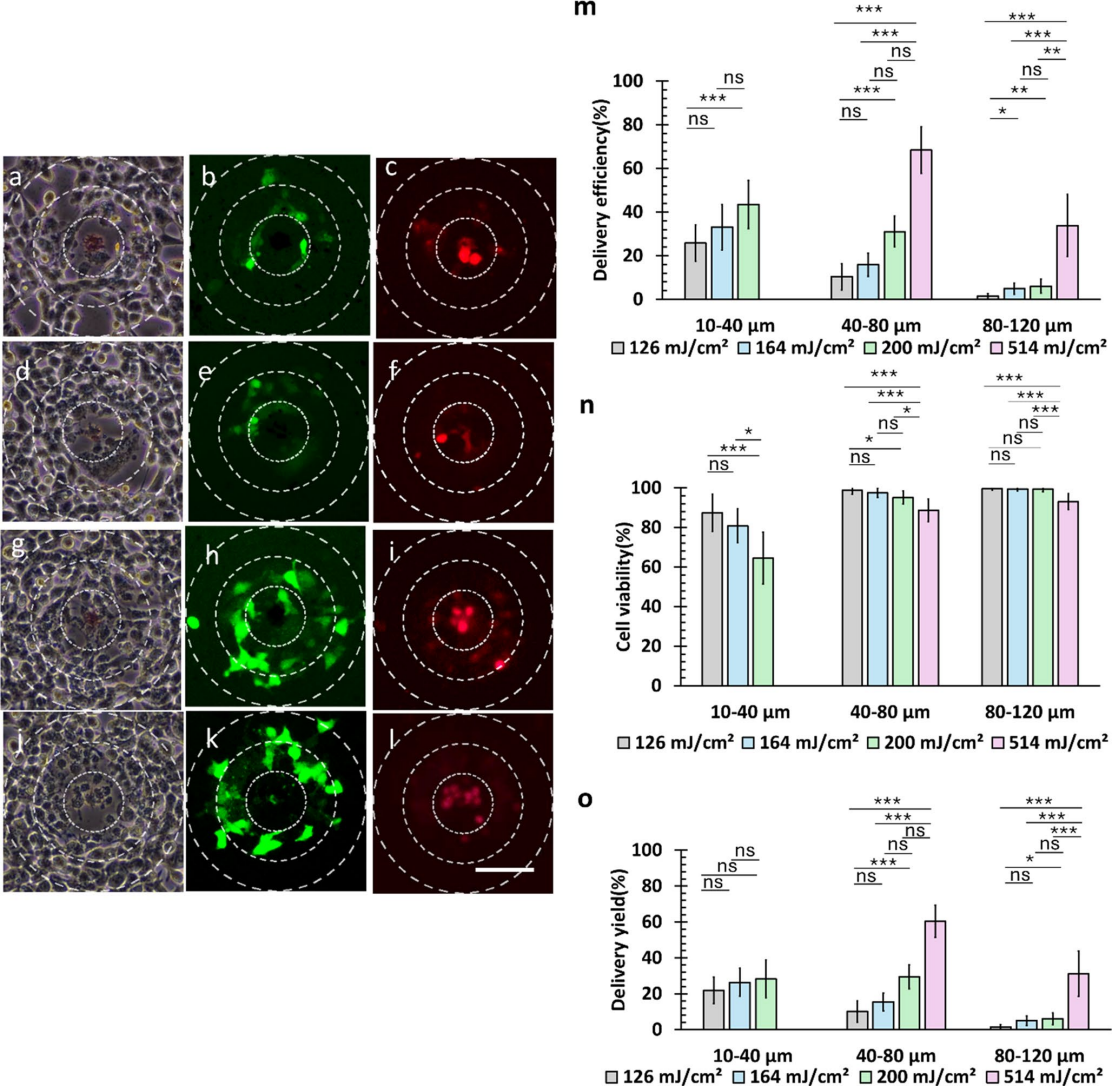
Optoporation of HEK-293 cells using a 20-µm diameter microdisk. (**a**–**c**) 126 mJ/cm², (**d**–**f**) 164 mJ/cm², (**g**–**i**) 200 mJ/cm², and (**j**–**l**) 514 mJ/cm². For each fluence: left column shows brightfield images, middle column shows FITC-positive cells (green, indicating successful delivery), and right column shows dead cells (red). Scale bar: 80 μm. (**m**) Delivery efficiency, (**n**) cell viability, and (**o**) delivery yield measured as a function of laser fluence and distance from the irradiation point

**Fig. 8:**
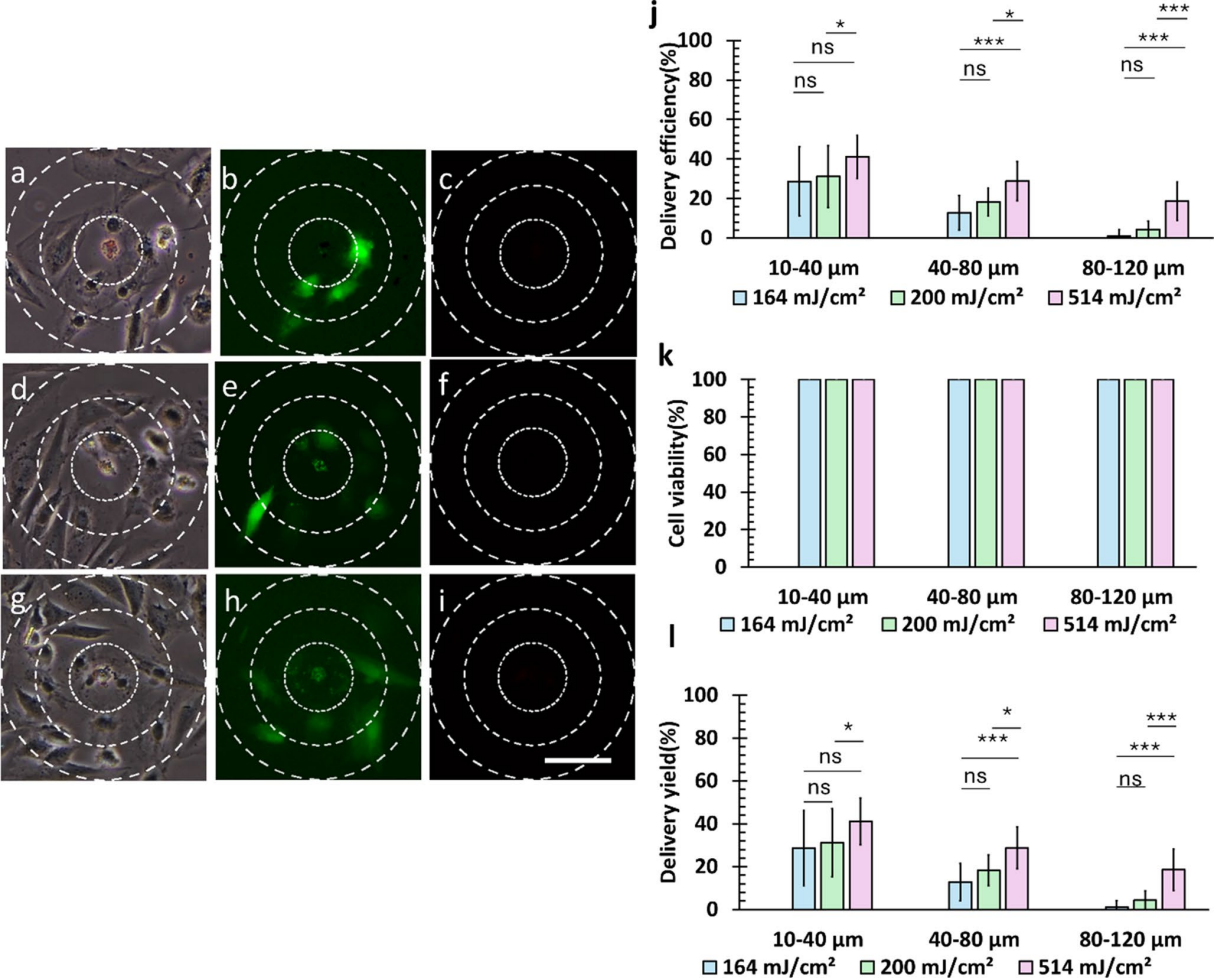
Optoporation of SAOS-2 cells using a 20-µm diameter microdisk. (**a**–**c**) 164 mJ/cm², (**d**–**f**) 200 mJ/cm², and (**g**–**i**) 514 mJ/cm². For each fluence, the left column shows brightfield images, the middle column shows FITC-positive cells (green, indicating successful delivery), and the right column shows dead cells (red). Scale bar: 80 μm. (**j**) Delivery efficiency, (**k**) cell viability, and (**l**) delivery yield measured as a function of laser fluence and distance from the irradiation point

The versatility of micropattern optoporation was investigated using 20-µm diameter disks to deliver FITC-dextran into different cell lines, comparing weakly adhering HEK-293 cells (Fig. 7) and SAOS-2 cells (Fig. 8) with HeLa cells as a reference. Due to their semi-adherent nature, HEK-293 cells demonstrated distinct responses in delivery efficiency (*η*_*D*_), cell viability (*η*_*V*_), and delivery yield (*η*_*Y*_) when exposed to laser pulses at various fluences. Cellular responses to 20 μm microdisk pattern irradiation at 514 mJ/cm^2^ laser fluence revealed variations among cell types (Supplementary Videos 3, 4 and 5), with HEK-293 cells showing extensive cell peeling and necrosis, while SAOS-2 cells exhibited reduced delivery effects compared to HeLa cells.

The delivery efficiency measurements, displayed in Fig. 7(m), demonstrated that laser and micropattern parameters affected cells differently based on their adhesion strength. HEK-293 cells exhibited increased susceptibility to necrosis and peeling at greater distances due to their physical properties, showing more pronounced effects compared to HeLa cells. As evidenced in Figs. 7(a), 7(d), 7(g), and 7(j), HEK-293 cells primarily experienced cell peeling and cell necrosis, attributable to their lower adhesion strength. The delivery efficiency consistently decreased with increasing radial distance, confirming the spatial extent of laser-induced effects.

The delivery yield analysis revealed distinct patterns at different radial distances from the irradiation point. At the highest laser fluence of 514 mJ/cm², extensive cell necrosis and peeling made evaluation impossible in the proximal 10–40 μm region, as evidenced in Figs. 7(j) and 7(k). Among lower fluences in this region, statistical analysis showed no significant differences in delivery yield (*p*_bonf_ = 1 for 200 mJ/cm² vs. 164 mJ/cm², *p*_bonf_ = 0.2 for 200 mJ/cm² vs. 126 mJ/cm², and *p*_bonf_ = 0.3 for 126 mJ/cm² vs. 164 mJ/cm²). Notably, at greater distances from the irradiation point, 514 mJ/cm² achieved the highest delivery yields despite compromised cell viability, reaching 60.3 ± 8.8% (*N*_T_ = 31 cells) in the 40–80 μm region and 31.7 ± 12.1% (*N*_T_ = 60 cells) in the 80–120 μm region.

Statistical analysis at equivalent laser energies (Figure S9) revealed significant differences for lower fluences (126 and 164 mJ/cm²) between the regions of 10–40 μm and 40–80 μm (*p*_*bonf*_ = 0.04 for both fluences) and between the 40–80 μm and 80–120 μm regions (*p*_*bonf*_ = 0.002 for both fluences). For higher energies of 200 mJ/cm^2^ and 514 mJ/cm^2^, significant differences were only observed between 40 and 80 μm and 80–120 μm regions (*p*_*bonf*_ < 0.001 for both fluences). These findings demonstrate that while lower laser fluences produced similar effects at shorter distances, higher fluences showed comparable effects at greater distances from the irradiation point, suggesting a distance-dependent relationship between laser fluence and delivery effectiveness.

Compared to HeLa cells, HEK-293 cells showed greater sensitivity to laser-induced effects at shorter distances. Significant delivery differences were observed even between proximal regions under low fluence, indicating that delivery in HEK-293 cells is more spatially localized and dependent on distance from the irradiation point. In contrast, HeLa cells exhibited broader effective delivery zones and higher tolerance to shockwave-induced stress, resulting in more uniform delivery across wider regions. These differences likely stem from variations in membrane composition, adhesion strength, and mechanical properties.

Fluorescence imaging of SAOS-2 cells during micropattern optoporation (Fig. 8) revealed their unique resistance to poration, reflected in both delivery efficiency and cell viability measurements. These cells demonstrated remarkable resilience, with neither cell necrosis nor detachment observed across all tested laser fluences, while maintaining 100% cell viability (*η*_*V*_) throughout all laser irradiation conditions. While no delivery was detected at 126 mJ/cm^2^, the delivery efficiency showed a consistent pattern: decreasing with increasing radial distance while increasing within the same radial distance as laser fluence increased.

The optimal delivery yield (*η*_*Y*_) was achieved at maximum laser fluences across all distances, with both delivery efficiency (*η*_*D*_) and delivery yield reaching their peak values. The measurements showed 41.0 ± 10.8% (average *N*_*T*_ = 4 cells) in the 10–40 μm range, declining to 28.7 ± 9.8% (average *N*_*T*_ = 14 cells) at 40–80 μm, and further decreasing to 18.6 ± 9.5% (average *N*_*T*_ = 22 cells) at 80–120 μm (Fig. 8l). These values were significantly higher than those observed at other laser fluences, demonstrating the effectiveness of higher laser fluences for delivery.

Statistical analysis (Figure S10) revealed significant differences in delivery yield between the 40–80 μm and 80–120 μm regions (*p*_bonf_ = 0.009 at 164 mJ/cm² and *p*_bonf_ = 0.001 at 200 mJ/cm²). The delivery yield showed a consistent decrease with increasing radial distance at any given energy level. At the highest laser fluence, significant differences emerged between the 10–40 μm and 40–80 μm regions (*p*_bonf_ = 0.04). These findings demonstrate that while lower laser fluences produced similar effects at shorter distances, higher fluences showed comparable effects at greater distances from the irradiation point, suggesting a distance-dependent relationship between laser fluence and delivery effectiveness.

Like the other cell types tested, delivery efficiency in SAOS-2 cells decreased with increasing radial distance from the laser. However, unlike those cells, SAOS-2 cells maintained high viability across all conditions. Statistical analysis confirmed that significant spatial differences in delivery yield still occurred, especially at higher fluences. Despite these differences, the consistently high viability makes SAOS-2 uniquely suited for high-intensity optoporation. This resilience distinguishes SAOS-2 from HeLa cells, which exhibit moderate tolerance and broader delivery zones, and HEK cells, which show greater sensitivity to short-range effects, highlighting cell-type-specific variations in shockwave response and membrane robustness.

## Discussion

### Novelty of methodology and setup

This study introduces a novel approach for investigating laser irradiated micropattern induced bioeffects on cell monolayers using modified substrates. The methodology enables the identification of multiple sites and facilitates long-term cell monitoring, overcoming significant challenges related to site-specific response faced in previous studies (Rau et al. 2006; Hellman et al. 2008) where laser was irradiated randomly irradiated on the substrate making the task of identifying the irradiation sites highly time consuming and tedious.

While laser irradiation has been extensively studied to optimize parameters for minimizing cell damage (Bergeron et al. 2015; Patskovsky et al. 2020), previous experiments without photoabsorbers faced difficulties in characterizing radial effects on cell monolayers. The identification of multiple sites and long-term cell monitoring posed challenges (Rau et al. 2006; Hellman et al. 2008). Our approach using modified substrates with micropatterns addresses these challenges by enabling precise identification of multiple irradiation sites and facilitating sustained observation of cellular responses.

### Cell-type specific responses

The cellular response to micropattern-generated bio-effects demonstrated significant variation across different cell types, with pattern size playing a crucial role in delivery outcomes. When using the 50-µm microdisk pattern, HeLa cells exhibited compromised delivery efficiency due to extensive cell detachment and necrosis throughout the 0–120 μm range. However, the 20-µm microdisk pattern generated smaller shockwaves that maintained delivery efficiency while minimizing cellular damage.

The investigation of multiple cell lines revealed that cellular adhesion strength significantly influenced the response to shockwave exposure. SAOS-2 cells demonstrated remarkable resilience when exposed to 20-µm microdisk-generated shockwaves, maintaining cellular integrity without experiencing necrosis or detachment. In stark contrast, HEK-293 cells, characterized by weaker adhesion properties, exhibited heightened sensitivity to the shockwaves, resulting in reduced delivery efficiency and diminished cell viability across an extended range. These findings highlight the importance of considering cell-specific characteristics when optimizing micropattern-assisted optoporation parameters.

### Mechanism analysis

Upon irradiation of a laser pulse on the micropattern, although visible plasma light emission (sparks) was not directly observed, laser-induced breakdown or localized plasma production below our detection threshold could still occur. Indeed, the main sources of laser-irradiated micropattern-induced bioeffects in this system are probably laser-induced breakdown and plasma generation on the microdisk. The PR-254/SU-8 surface is rapidly heated by the 532-nm, 5-ns laser pulse, which causes a small layer of water at the interface to evaporate and then explode into bubbles. Although we cannot define a precise breakdown threshold, the onset of bubble formation could be considered a practical threshold for significant material modification. The mechanism likely involves rapid heating of the micropattern surface, vaporization of a thin water layer, and subsequent bubble nucleation and growth, which would contribute to the observed bioeffects.

Our nanosecond pulsed laser study revealed that micropattern size and laser fluence significantly influence cellular responses through three primary mechanisms affecting membrane integrity. Larger micropatterns contributed to increased cell necrosis and detachment over greater distances, impacting delivery efficiency in a cell type-specific manner. The analysis of these mechanisms is crucial for understanding the observed changes in cell membrane integrity: (a) Expansion and collapse of a cavitation bubble, (b) Laser induced shockwave upon micropattern irradiation, and (c) Heat transfer between the micropattern and cells.

The first mechanism involves rapid photothermal absorption upon laser irradiation of micropatterned surfaces, leading to cavitation bubble formation through optical breakdown and vaporization. These bubbles undergo explosive expansion and subsequent collapse, generating high velocity microjets that exert significant mechanical stress on nearby cell membranes (Zhou et al. 2012; Long et al. 2020; Yasukuni et al. 2022). This process results in transient poration and enhanced membrane permeability through the mechanical forces generated during bubble dynamics.

At shorter timescales, the second mechanism occurs when PR-254/SU-8 surfaces experience rapid heating from the 532-nm, 5-ns laser pulse, inducing a phase change in the adjacent water layer. This generates supersonic shock waves, whose propagation creates transient stress fields in the surrounding medium, causing membrane deformation and temporary disruption of the phospholipid bilayer structure (Noack and Vogel 1998; Gomez Godinez et al. 2021).

The third mechanism involves localized thermal effects, where heat transfer from micropatterns leads to the formation of short-lived hydrophilic pores through lipid bilayer denaturation, enhancing membrane permeability to various molecules (Qian et al. 2020). These mechanisms collectively depend on the interaction between the target surface and laser irradiation, with larger micropatterns producing more pronounced bioeffects on the cell monolayer while smaller micropatterns cause less bioeffects.

Given the complexity of these rapid physical phenomena occurring at very short time scales, further investigation using advanced techniques such as time-resolved spectroscopy, high-speed imaging, and molecular dynamics simulations would enhance our understanding of the induced bioeffects, ultimately enabling optimized designs for specific applications in biophotonic cell manipulation.

## Conclusions

A quantitative distance-dependent method was developed to evaluate laser-irradiated micropattern-induced bioeffects on intracellular delivery using pigmented SU-8 microdisks. This approach enabled precise control of both microdisk size and laser fluence while allowing direct observation of cellular responses at varying radial distances from the irradiation point. The effectiveness of the bioeffects was significantly influenced by two key factors: micropattern size and cell-type-specific characteristics.

The study revealed distinct responses between different cell types and pattern sizes. Larger micropatterns led to increased cell necrosis and detachment over broader ranges, while smaller micropatterns maintained comparable delivery efficiency with minimal cellular damage. Cell-specific properties played a crucial role in treatment outcomes, as demonstrated by the contrasting responses between SAOS-2 and HEK-293 cells. While SAOS-2 cells maintained their integrity throughout the treatment, HEK-293 cells showed increased susceptibility to detachment and necrosis over larger distances.

This systematic analysis establishes a framework for optimizing laser and micropattern parameters in micropattern-assisted optoporation. The method offers advantages over conventional approaches through precise spatial control and real-time monitoring capabilities. These findings enhance our understanding of shockwave-mediated intracellular delivery mechanisms and provide practical guidelines for developing more effective therapeutic delivery strategies while preserving cell viability.

## Supplementary Information

Below is the link to the electronic supplementary material.

Supplementary Material 1

## Data Availability

Data will be made available upon reasonable request.
