# Supplementary material for: Distance-dependent spatial analysis of micropattern-generated shockwave for cell-type specific intracellular delivery
Source: Biomed Microdevices. 2025 Jun 23;27(3):30. doi: 10.1007/s10544-025-00758-x (PMC12183134; doi:10.1007/s10544-025-00758-x)
Supplement: Supplementary file 1 — Supplementary Material 1 [file 10544_2025_758_MOESM1_ESM.docx]

**Distance-Dependent Spatial Analysis of Micropattern-Generated Shockwave for Cell-Type Specific Intracellular Delivery**

Aniket Mishra^1^, Shunya Okamoto^1^, Takayuki Shibata^1,5^, Tuhin Subhra Santra^2^,
Sangjin Ryu^3,4^, Moeto Nagai^1,5^*

^1^Department of Mechanical Engineering, Toyohashi University of Technology, Japan

^2^Department of Engineering Design, Indian Institute of Technology, Madras, India

^3^Department of Mechanical and Materials Engineering, University of Nebraska-Lincoln, USA

^4^Nebraska Center for Materials and Nanoscience, University of Nebraska-Lincoln, USA

^5^Institute for Research on Next-generation Semiconductor and Sensing Science (IRES²), Toyohashi University of Technology, Japan

*Corresponding author: nagai@me.tut.ac.jp

**Silicone wall fabrication for microchips:**

A 1-mm-thick silicone rubber sheet was cut according to the required dimensions.

**
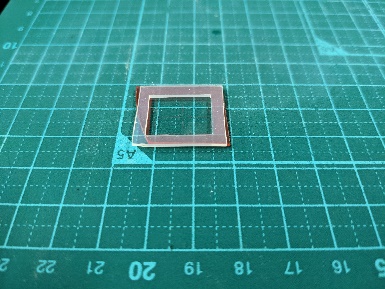
**

**Supplementary Figure S1:** Silicone wall structure for the microchip.

b


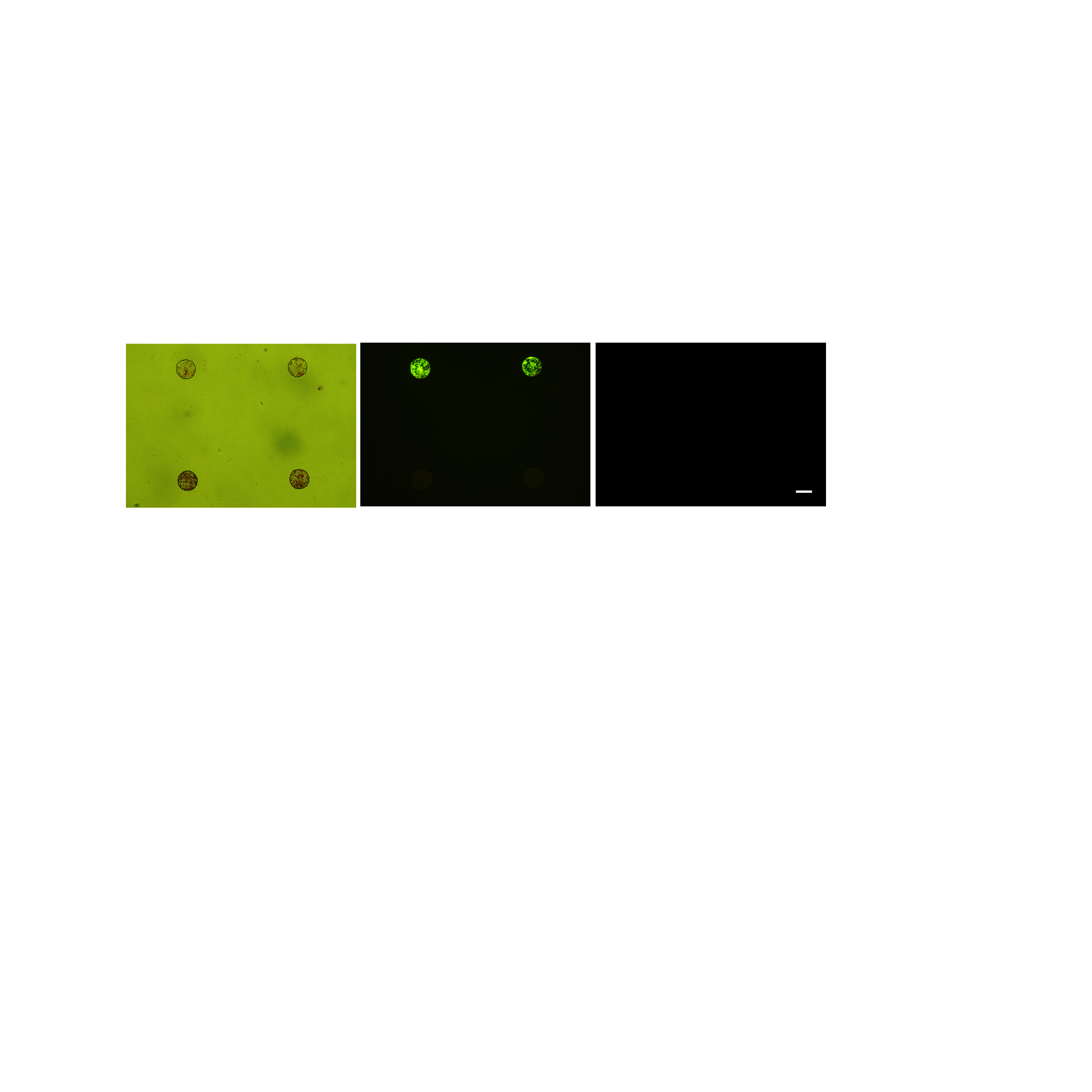


c

a

**Supplementary Figure S2: Fluorescence induced in the micropattern structure upon laser irradiation. Scale bar: 50 µm. (a) Brightfield image (b) FITC filter image (c) PI filter image**


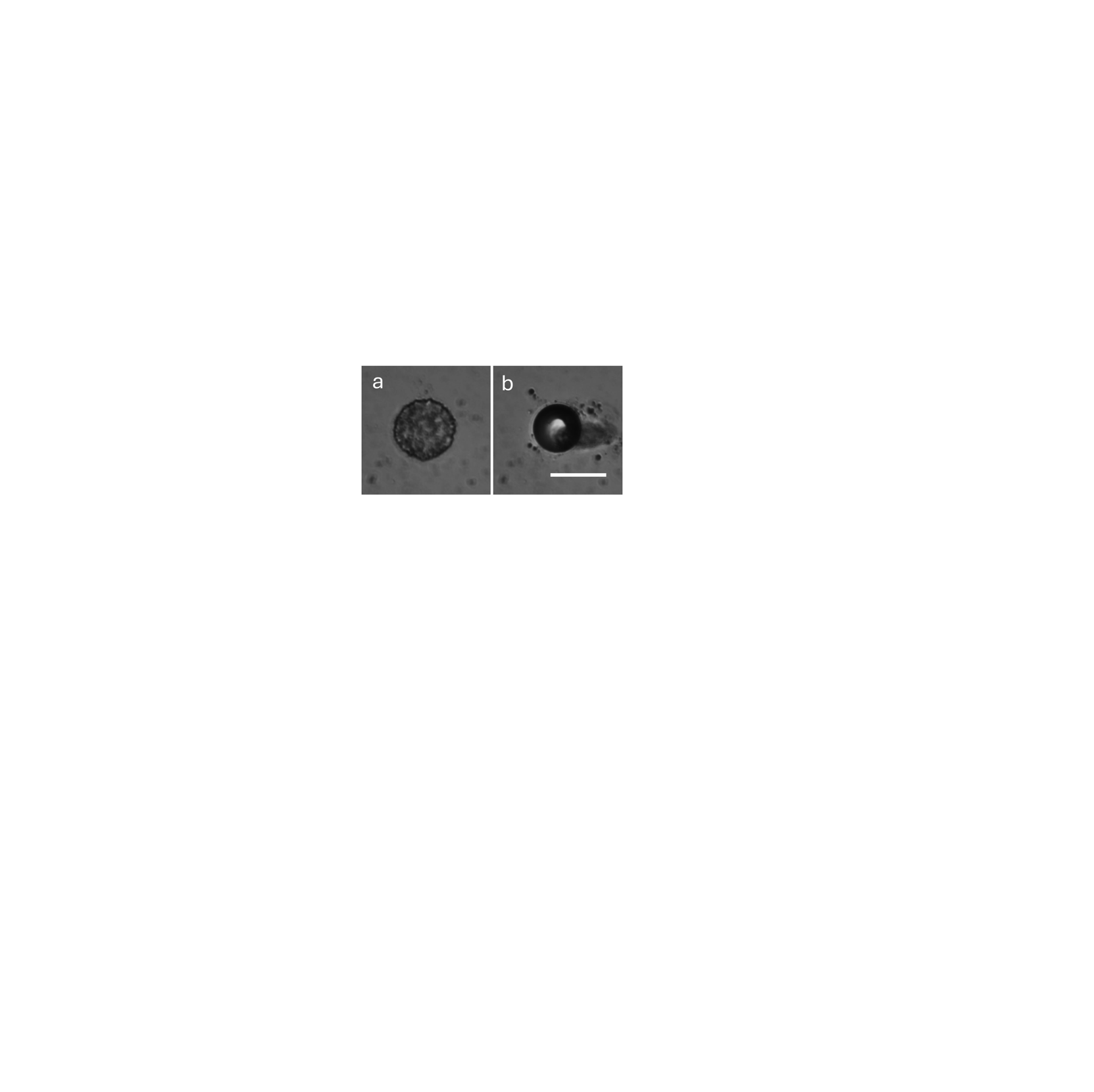


**Supplementary Figure S3: Micropattern removal at a laser fluence of 514 mJ/cm^2^. Scale bar is 50 µm. (a) Micropattern before laser irradiation (b) Micropattern after laser irradiation.**


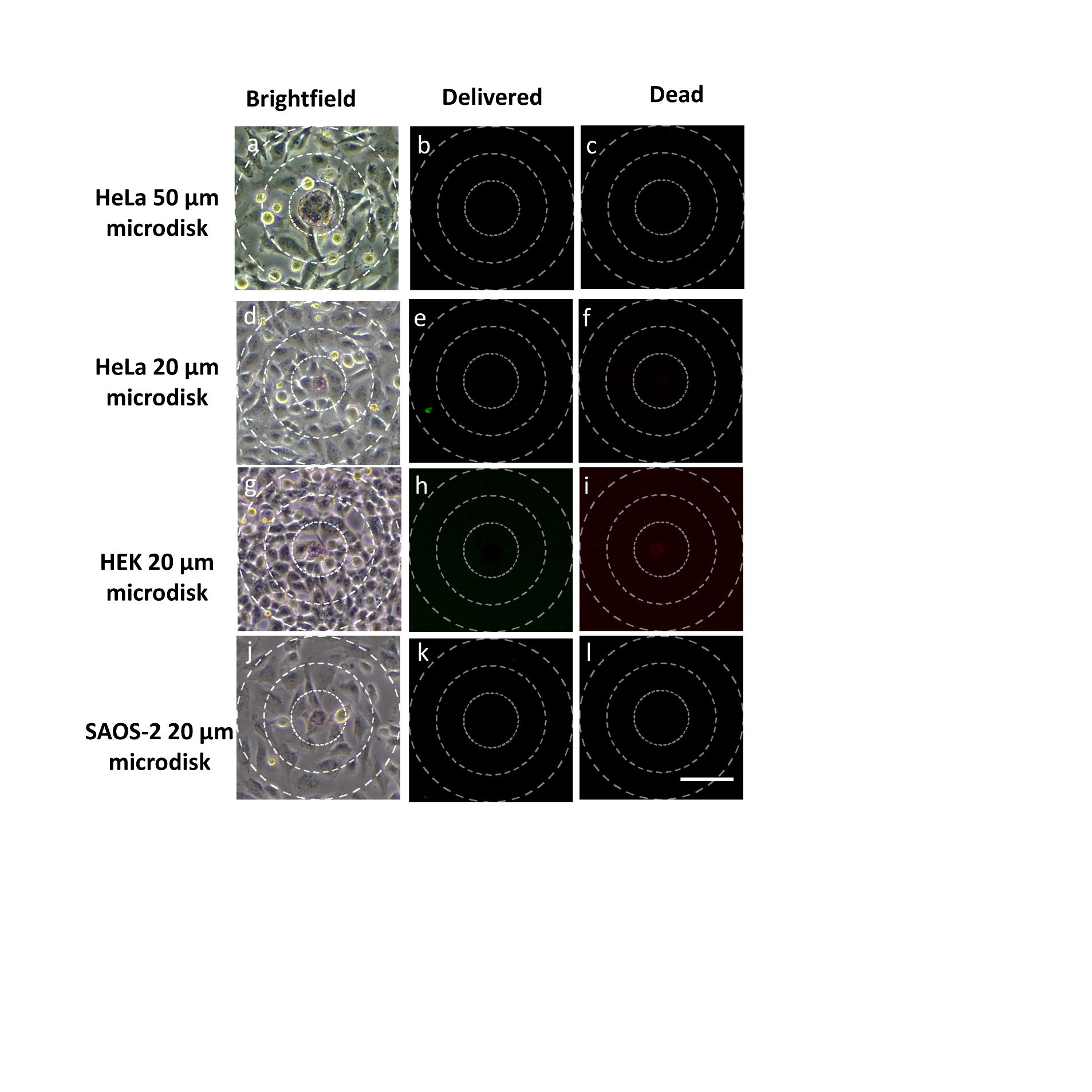


**Supplementary Figure S4: Micropattern control images of cells without laser irradiation. (Scale bar is 80 µm)(a)– (c) HeLa cells with a 50-µm microdisk (d)– (f) HeLa cells with a 20-µm microdisk (g)– (i) HEK cells with a 20-µm microdisk (g)– (i) SAOS-2 cells with a 20-µm microdisk.**


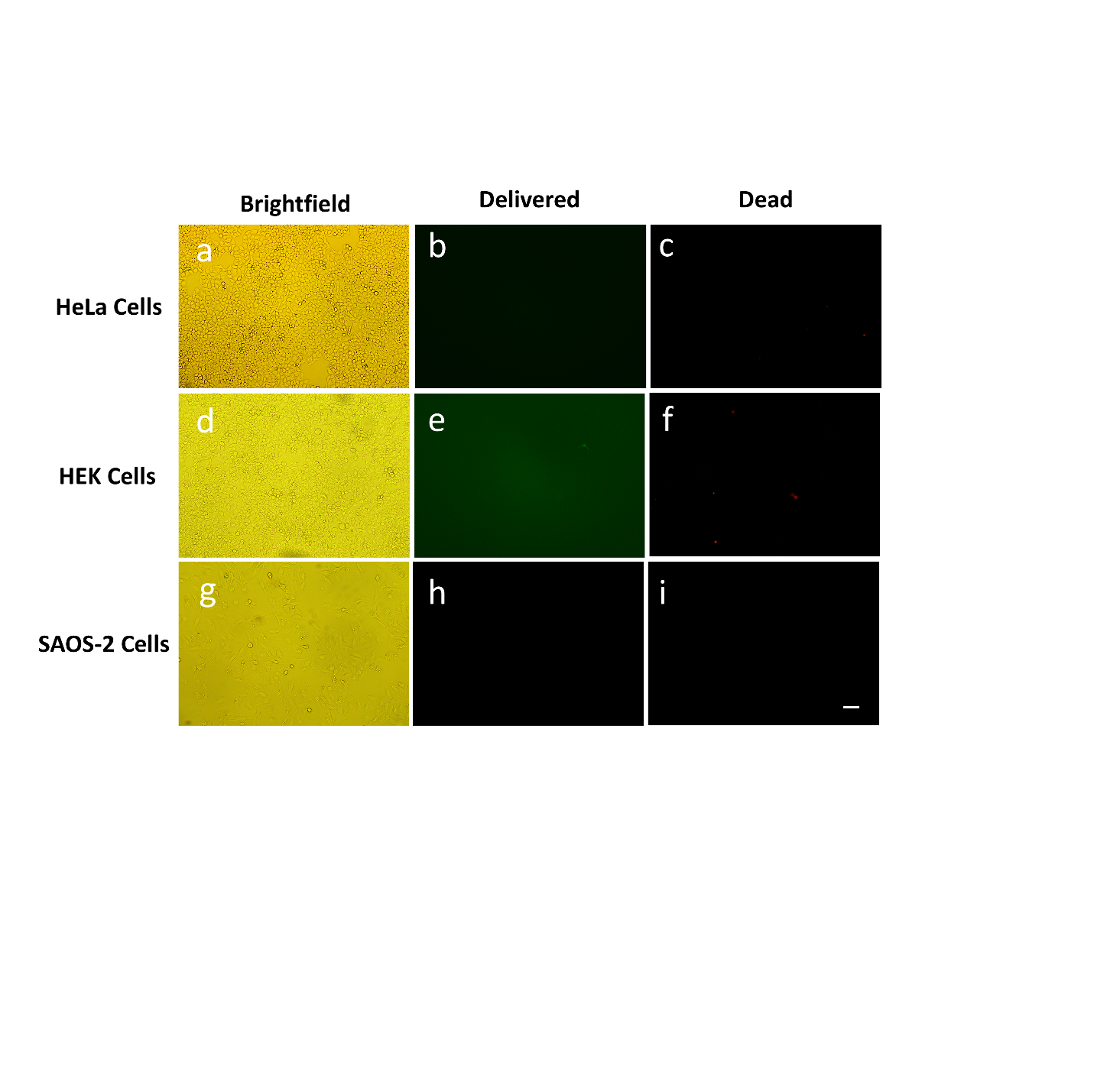


**Supplementary Figure S5: Laser control images of all the cells at 514mJ/cm^2^  without micropattern on glass substrate. Scale bar: 50 µm.**

In the given images of HeLa, HEK-293, and SAOS-2 cells cultured on the glass substrate single pulse of laser was irradiated at every 200 µm distance at a laser fluence of 514mJ/cm^2^.


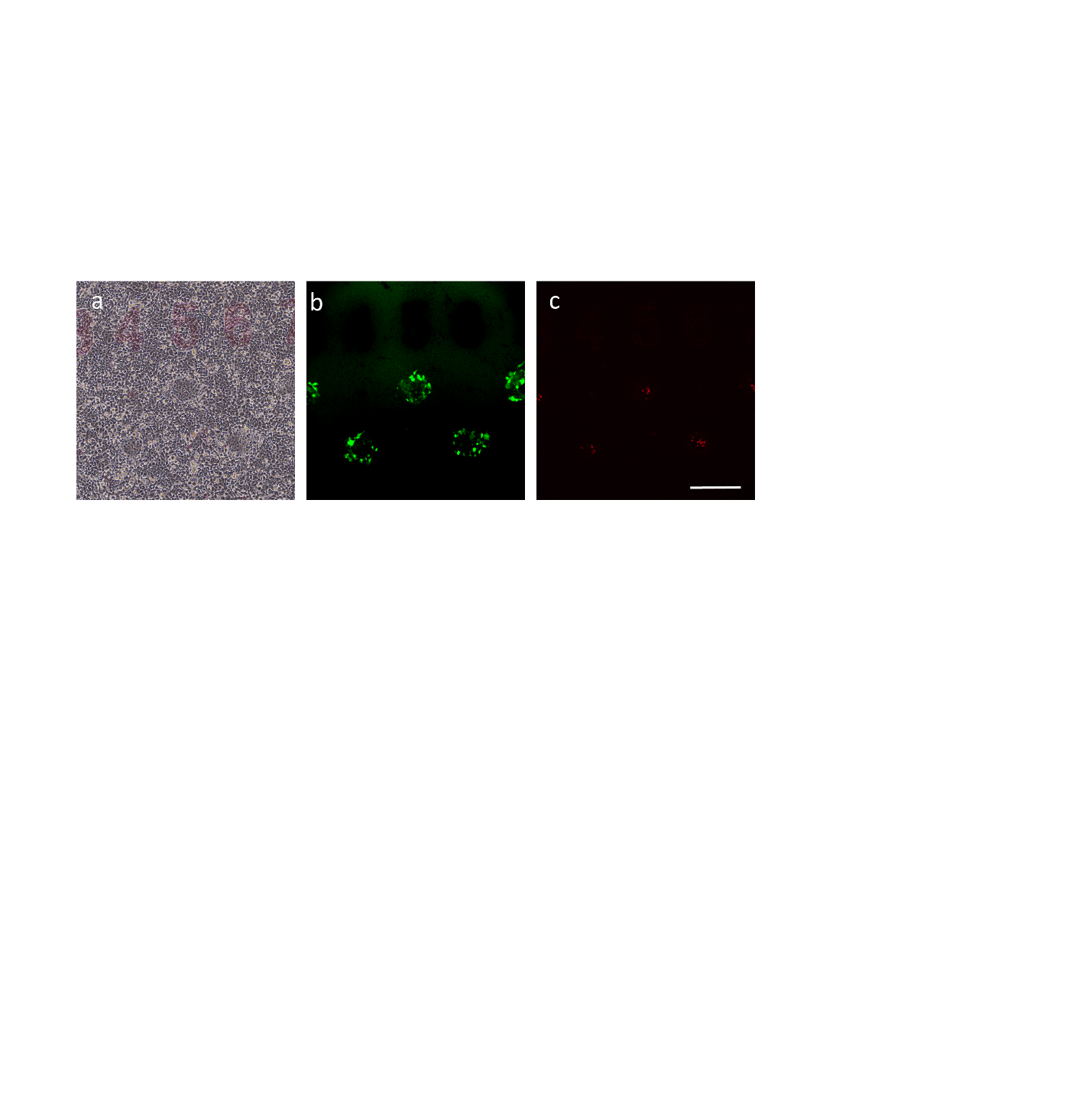


**Supplementary Figure S6: Site-specific Opotoporation ion HEK cells. Scale bar: 200 µm). Intracellular delivery was observed only around laser Irradiated micropatterns at 514 mJ/cm^2^ in the first row and at 200 mJ/cm^2^ in the second row.**


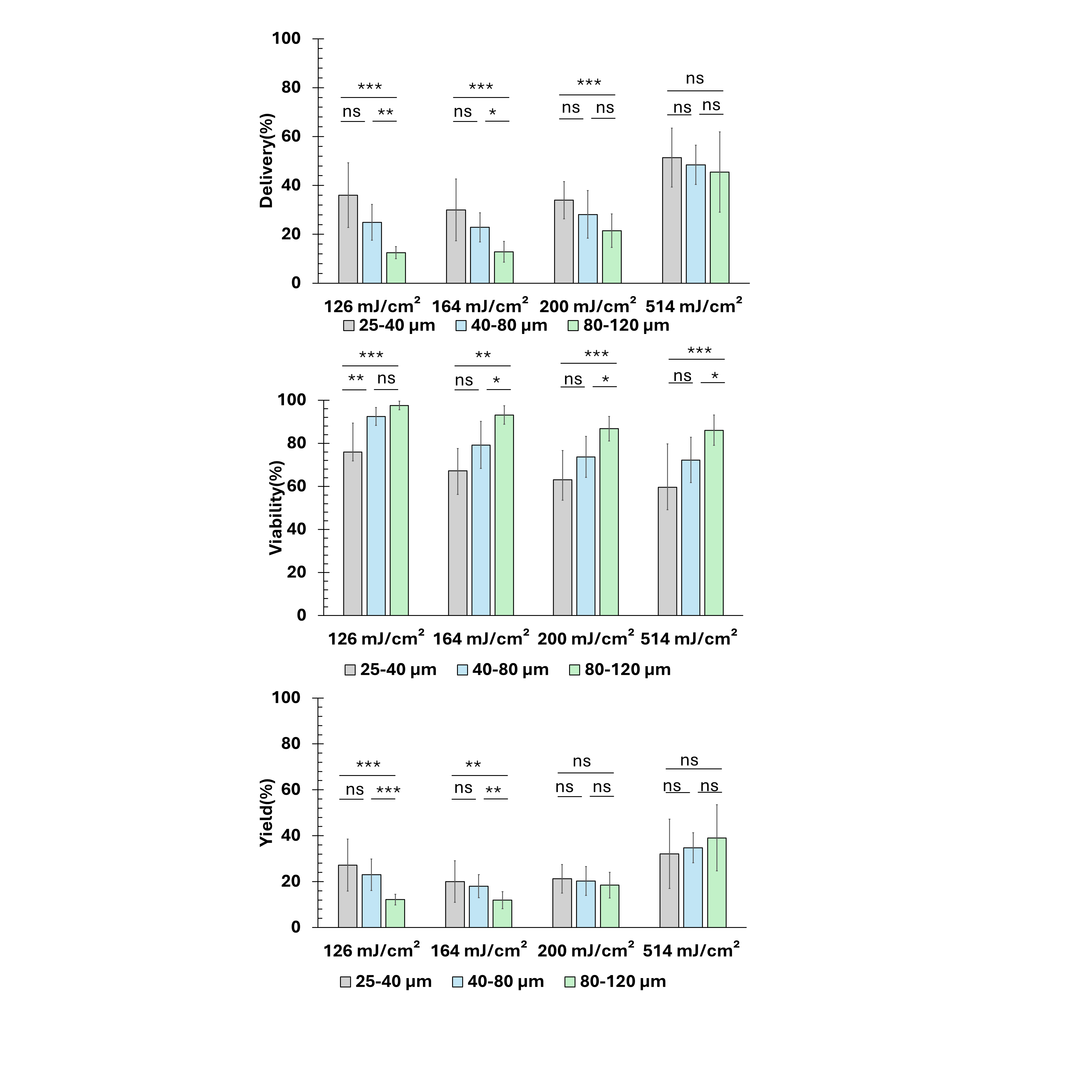


c

b

a

**Supplementary Figure S7:** Distance-dependent molecular delivery characteristics using 50 µm micropattern for HeLa cells. (a) Delivery efficiency, (b) cell viability, and (c) delivery yield as a function of laser fluence and distance from the irradiation point.

**
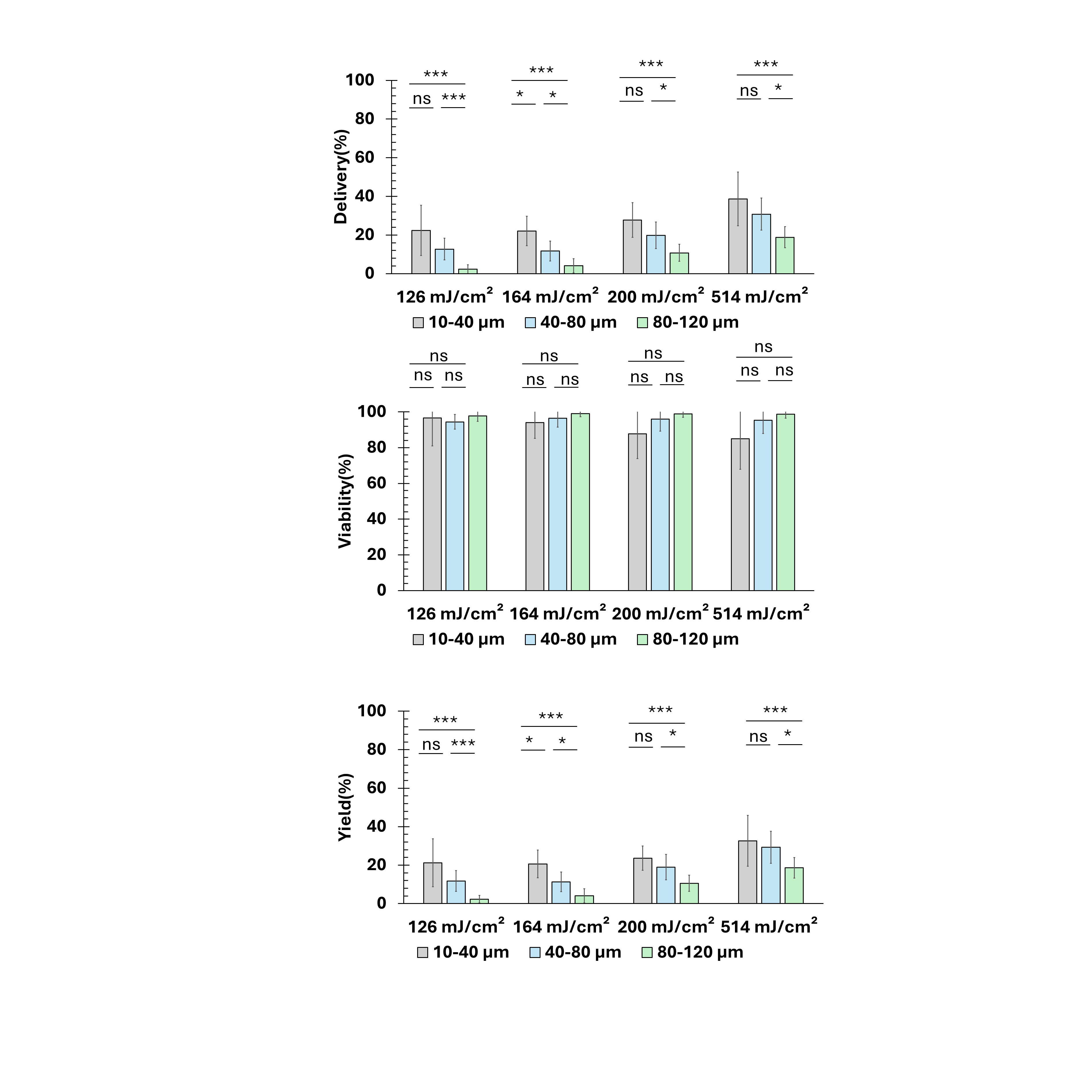
**

c

b

a

**Supplementary Figure S8:** Distance-dependent molecular delivery characteristics using 20 µm micropattern for HeLa cells. (a) Delivery efficiency, (b) cell viability, and (c) delivery yield as a function of laser fluence and distance from the irradiation point.


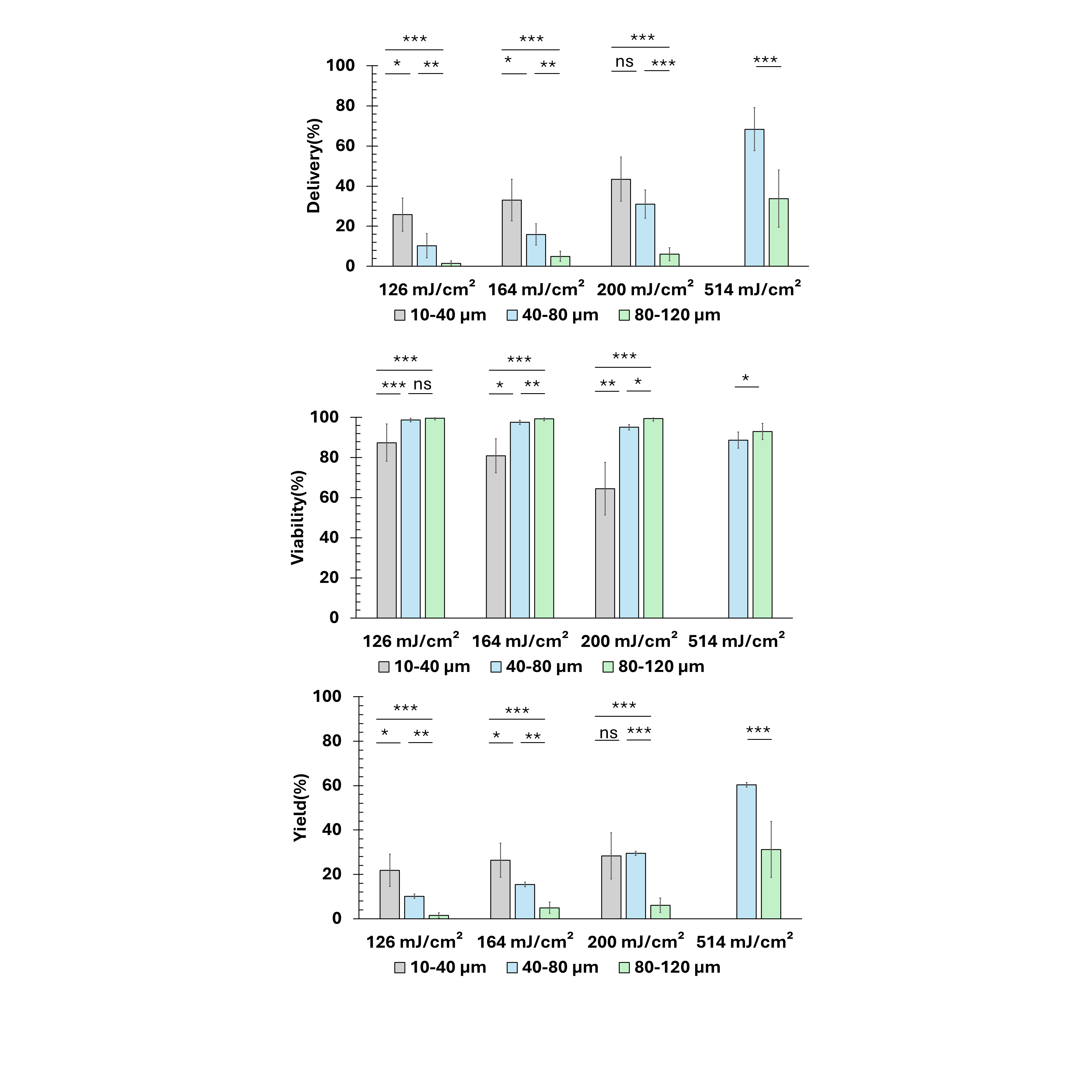


c

b

a

**Supplementary Figure S9:** Distance-dependent molecular delivery characteristics using 20 µm micropattern for HEK-293 cells. (a) Delivery efficiency, (b) cell viability, and (c) delivery yield as a function of laser fluence and distance from the irradiation point.


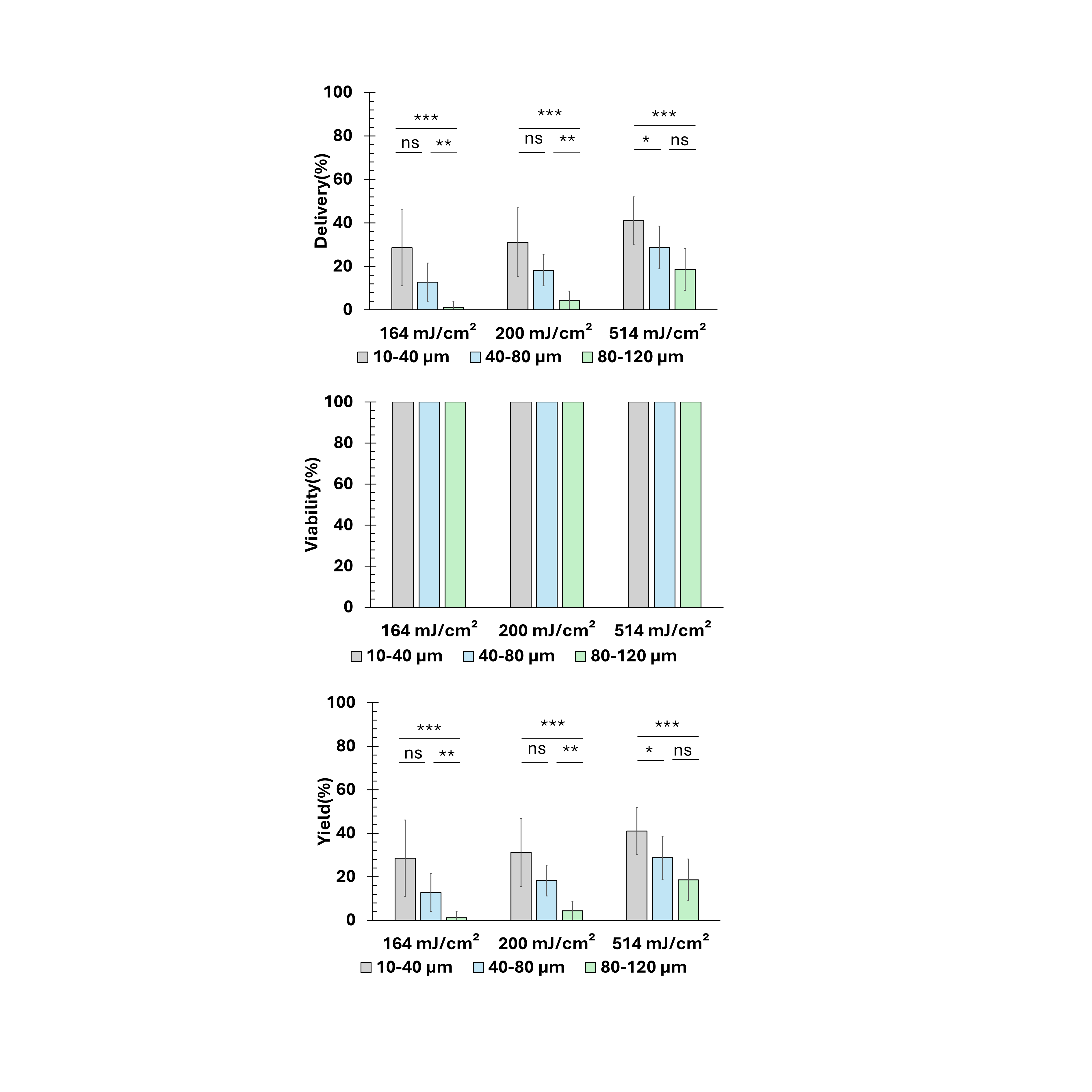


a

b

c

**Supplementary Figure S10:** Distance-dependent molecular delivery characteristics using 20 µm micropattern for SAOS-2 cells. (a) Delivery efficiency, (b) cell viability, and (c) delivery yield as a function of laser fluence and distance from the irradiation point.
